# Inhibitory Activity of Calcium and Sodium Ion Channels of Neurotoxic *Protoplaythoa variabilis* V-Shape Helical Peptide Analogs and Their Neuroprotective Effect In Vitro

**DOI:** 10.3390/ph18111701

**Published:** 2025-11-10

**Authors:** Ariane Teixeira dos Santos, Victoria Jiménez Carretero, Jorge Hernández Campano, Ricardo de Pascual, Nan Xu, Simon Ming-Yuen Lee, Clarence Tsun Ting Wong, Gandhi Rádis-Baptista, Jesús Miguel Hernández-Guijo

**Affiliations:** 1 Graduate Program in Pharmaceutical Sciences, Faculty of Pharmacy, Dentistry and Nursing, Federal University of Ceará, Fortaleza 60430-370, Ceara, Brazil; 2 Department of Pharmacology and Therapeutics, Faculty of Medicine, Universidad Autónoma de Madrid, 28049 Madrid, Spain; 3 Department of Food Science and Nutrition, The Hong Kong Polytechnic University, Hung Hom, Hong Kong SAR, China; 4 Department of Applied Biology and Chemical Technology, The Hong Kong Polytechnic University, Hong Kong SAR, China; 5 Laboratory of Biochemistry and Biotechnology, Institute for Marine Sciences, Federal University of Ceará, Fortaleza 60430-370, Ceara, Brazil; 6 Department of Biochemistry and Biophysics, Institute for Health Sciences, Federal University of Bahia, Salvador 40231-300, Bahia, Brazil; 7 Ramón y Cajal Institute for Health Research, IRYCIS, Hospital Ramón y Cajal, 28034 Madrid, Spain

**Keywords:** cnidarian peptide, *Protopalythoa variabilis*, PpVα peptides, chromaffin cells, cellular excitability, calcium channels, sodium channels

## Abstract

**Background/Objectives:** One of the neurotoxic components from the sea trumpet polyps, *Protopalythoa variabilis* (Cnidaria, Anthozoa), is a 26-residue, V-shape helical peptide (PpVα). Its synthetic versions, i.e., the linear, the single-disulfide-bonded analog, and the chimeric peptide with a 6-residue stretch of the N-terminal native homologous peptide covalently linked to the linear sequence, were investigated for their activity on ion channels responsible for cellular excitability and synaptic transmission. **Methods:** Molecular docking analyses and dynamic simulations focused on the ability of PpVα peptides to bind ion channels selectively through interaction with critical residues at their binding sites. **Results:** Electrophysiological studies using the patch clamp technique with sympathetic bovine chromaffin cells from the adrenal medulla confirmed that PpVα analogs can block both sodium and calcium currents, which are responsible for initiating and propagating action potentials, respectively, and for neurotransmitter release. Additionally, the peptides displayed neuroprotective effects, attenuating cellular damage induced by veratridine, which interferes with sodium channel activity, and by oligomycin and rotenone (O/R), which affect mitochondrial function. **Conclusions:** The block of calcium and sodium channels and the neuroprotective effects against oxidative stress make the PpVα peptide scaffold an attractive template for developing agents that has significant clinical potential in several areas, such as the treatment of neurological diseases (epilepsy, multiple sclerosis, and neurodegenerative diseases), neuroprotection in acute events (stroke and traumatic brain or spinal cord injuries), the management of neuropathic pain, the prevention of ischemic damage, and psychiatric disorders (anxiety and bipolar disorder).

## 1. Introduction

Cnidarian venoms are rich sources of peptides that essentially act by disturbing the cellular homeostasis (e.g., pore-forming toxins) [1,2] or by interfering with ion channels (neurotoxic peptides or neurotoxins) [3], affecting cell survival and neurotransmission. Cnidaria comprises a large phylum, mainly marine species, that includes hydra, Portuguese Man-O-War, fire corals, and hydras (Hydrozoa), jellyfish (Scyphozoa), sea wasps (Cubozoa), sessile medusae (Staurozoa), and the true corals, sea anemones, zoanthids, and sea fans (Anthozoa) [4]. They all have a unique venom organelle (nematocyst) that, upon chemical stimuli or osmotic pressure, ejects a harpoon-like structure that discharges a cocktail of toxic components, causing envenomation and paralysis of the attackers and prey [5].

For decades, cnidarian toxins have sparked the scientific community’s interest in characterizing their venom components, obtaining selective pharmacological probes, and developing therapeutic leads [6,7,8]. An example of a cnidarian toxin converted into a drug lead is dalazatide (ShK-186) [9]. The Shk-186 is a synthetic peptide analog derived from the sea anemone *Stichodactyla helianthus* and is currently in development as a therapeutic agent for the treatment of autoimmune diseases and rheumatoid arthritis. ShK is a potent blocker of voltage-dependent potassium ion-channel subtype 1.3 (Kv1.3) [10].

The most-studied cnidarian venoms are those of sea anemones and jellyfish. The repertoires of cnidarian toxins across various species and the diversity of peptide structures have advanced further with the advent of omics sciences [11,12,13,14,15,16]. The transcriptome of the understudied brown trumpet polyp *Protopalythoa variabilis* revealed one of these toxin cocktails from a less-studied cnidarian species. Among several classes of peptide toxins, such as neurotoxic peptides, hemostatic and hemorrhagic toxins, membrane-active (pore-forming) proteins, protease inhibitors, mixed-function venom enzymes, and venom auxiliary proteins, we disclosed the sequence of a unique V-shape α-helical peptide (PpVα) [17]. The only counterpart known of PpVα is its homologous peptide that inhibits voltage-gated calcium and potassium channels of the superior cervical ganglion neurons of the rat, and it is present in the tissue of the *P. variabilis* sister species, *Palythoa caribeaorum* [18].

Previously, we investigated the synthetic versions of the linear PpVα and the folded PpVα analog, which contains an internal single disulfide bond, and the linear, chimeric PpVα. We found that folded and chimeric PpVα peptides could be effective blockers of voltage-gated sodium channels in vertebrates. Moreover, in vivo, in the zebrafish model of epilepsy, folded PpVα displayed antiepileptic and neuroprotective activities by reversing the 6-hydroxydopamine (6-OHDA)-induced neurotoxicity on zebrafish locomotor behavior and, significantly, preventing the 6-OHDA-induced excessive ROS generation and subsequent dopaminergic neuron loss [14].

Voltage- and ligand-gated ion channels are involved in numerous physiological cellular functions, including neurotransmission, muscle contraction, insulin secretion, water transport across membranes, and lymphocyte activation [19]. In contrast, dysfunctions of ion channels are involved in severe, chronic neurodegenerative disorders like Alzheimer’s, Parkinson’s, and Huntington’s diseases, epilepsy, schizophrenia, neuropathic pain, multiple sclerosis, amyotrophic lateral sclerosis, age-related disorders, and brain tumors [20,21]. Besides ion channel dysfunction, molecular alterations that contribute to the progression of neurodegenerative disease include inflammation and oxidative stress [22]. Despite the advances in the comprehension of the molecular mechanisms of neurodegenerative disorders, their management is often disease-specific, aiming to relieve the degradative symptoms in patients [23]. Thus far, studying highly selective and potent peptides as pharmacological agents and drug leads that act on ion channels and neural receptors can continuously benefit drug development and clinical medicine.

Herein, we report the electrophysiological activity of PpVα peptide analogs on calcium, potassium, and sodium currents in a model of the sympathetic neuron, specifically, the bovine chromaffin cells from adrenal medullae, as well as their presumed cytotoxicity and neuroprotection in vitro. Patch clamp records indicated that PpVα peptide analogs block calcium and sodium ion channels preferentially, as indicated by peptide-ion channel interaction energy calculation through molecular docking and dynamic simulation. The peptides displayed a neuroprotective effect in cultured neuronal cells, reversing the toxic effects of veratridine—an activator of the sodium-ion channel- and of a combination of the mitochondrial disruptors, i.e., oligomycin and rotenone.

Therefore, this study was designed to investigate the electrophysiological and biological activities of synthetic analogs of the PpVα peptide derived from *Protopalythoa variabilis*. Specifically, we aimed to characterize their effects on voltage-gated sodium, calcium, and potassium channels in a neuronal model and to assess their potential cytotoxic and neuroprotective properties in vitro. We hypothesized that structural modifications of the PpVα peptide would differentially influence its affinity and selectivity toward specific ion channel subtypes, and that such modulation could underlie protective mechanisms against neurotoxic insults associated with oxidative stress and mitochondrial dysfunction.

## 2. Results

### 2.1. Three-Dimensional Structure Modeling and Molecular Dynamics Simulation of PpVα Peptide Analogs

**Figure 1:**
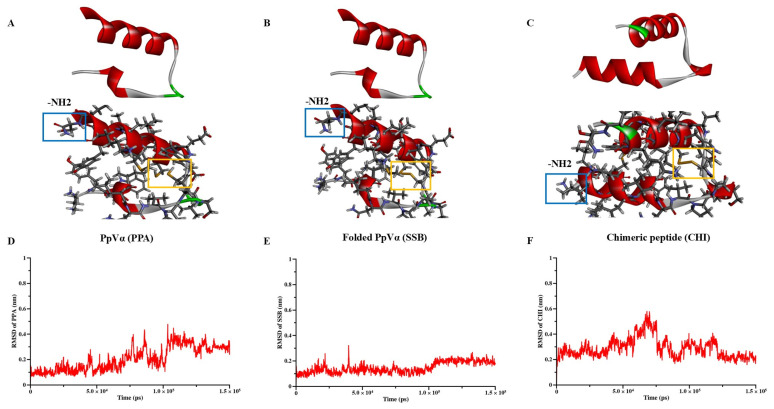
Three-dimensional structure modeling and molecular dynamics simulation of PpVα peptide analogs. (**A**–**C**) Representations of modeled structures of PPA, SSB, and CHI. The blue square frame represents the regions of C-terminal amidation, and the cysteine residues and disulfide bonds are in the yellow square frame. (**D**–**F**) The backbone RMSD of all atoms of PPA, SSB, and CHI.

Based on the peptide sequences provided in Table 1, AlphaFold2 was utilized to construct the 3D structures of the PpVα peptide analogs. The structures with the highest confidence scores were selected for further analysis. Subsequently, CHARMM-GUI was used to perform C-terminal amidation and to establish disulfide bonds (Figure 1A–C). Molecular dynamics simulations were conducted to analyze the stability of the modified structures. The root-mean-square deviation (RMSD) values were calculated based on the backbone atoms of the peptides. As depicted in Figure 1D–F, the RMSD values for PPA, SSB, and CHI plateaued at 0.25 nm, 0.15 nm, and 0.20 nm, respectively. These results demonstrated that all structures were stable and suitable for subsequent experiments, with the SSB structure being the most stable.

### 2.2. Molecular Docking of PpVα Peptide Analogs and Sodium and Calcium Ion Channels

**Figure 2:**
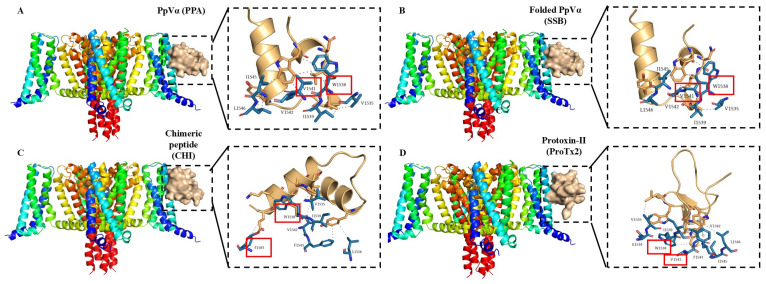
Docking poses and the corresponding zoom representations of the Na^+^-channel interacting with PpVα peptide analogs and ProTx2. (**A**) PPA; (**B**) SSB; (**C**) CHI; (**D**) ProTx2. The red square frame highlights the critical amino acids in the binding with the Na^+^-channel receptor.

**Figure 3:**
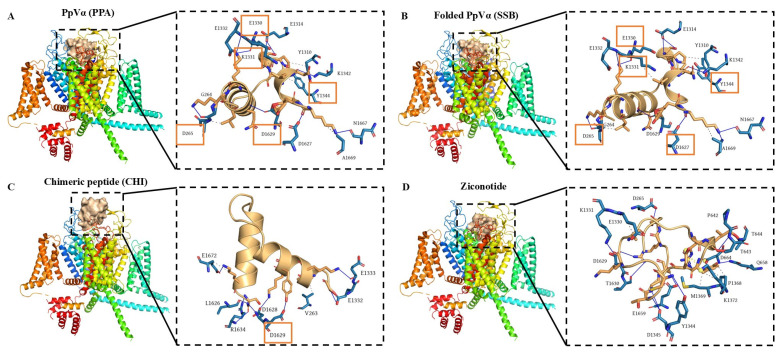
Docking poses and the corresponding zoom representations of the Ca^2+^-channel interacting with PpVα peptide analogs and ziconotide. (**A**) PPA; (**B**) SSB; (**C**) CHI; (**D**) Ziconotide. The orange box highlights the amino acids in the PpVα peptide analogs that are the same as those in ziconotide when binding to the Ca^2+^-channel.

Molecular docking was performed using ZDOCK. Since lower docking scores indicate stronger affinity and higher complex stability, complexes docked in the active pocket with the lowest scores were selected. The protoxin-II (ProTx2) from the Peruvian green velvet tarantula is a selective antagonist of the human Na_v_ channels [24], and ziconotide, derived from the ω-conotoxin MVIIA, is a highly selective and potent inhibitor of Ca_v_ channels [25]. Therefore, both were used as a reference in the docking study. The docking scores for the interactions of the Na^+^-channel with PPA, SSB, and CHI were −63.40, −63.40, and −62.06, respectively, indicating similar affinities for the Na^+^-channel and only slightly less potent than ProTx2 (−68.80). Regarding the virtual interaction with the Cav channel, PPA, SSB, and CHI exhibited lower docking scores of −80.61, −80.61, and −82.70, respectively, and were considerably more potent than ziconotide (−168.67). These values indicate that the Na^+^-channel is the preferred target for these peptides. According to previously reported literature [26], W1538, V1541, F1583, R1602, and Y1537 are key binding residues inhibiting the Na_v_ channel function. As expected, PPA and SSB, like ProTx2, bound to the key residues V1541 and W1538, while CHI interacted with F1583 and W1538 (Figure 2A–D). In addition, in the molecular docking with the Ca_v_ channel (Figure 3A–D), using the binding site of the positive control ziconotide as a reference, both PPA and SSB shared multiple similar amino acid interactions with ziconotide, specifically at E1330, K1331, Y1344, D1629, and D265. In contrast, CHI only shared one common interaction site at D1629. These amino acids may play crucial roles in the inhibition of Ca_v_ channels by these peptides.

### 2.3. Molecular Dynamics Simulation of PpVα Peptide Analogs and Sodium and Calcium Ion Channels

**Figure 4:**
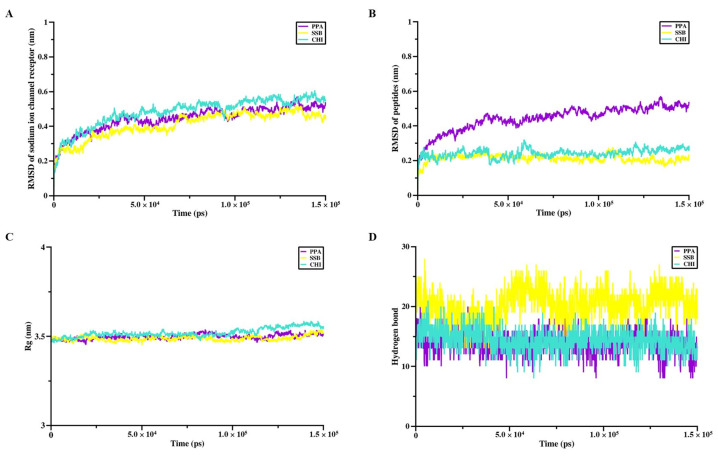
Molecular dynamics simulation between the Na^+^-channel and the PpVα peptide analogs. (**A**) RMSD value of Na_v_ channel receptor during molecular dynamics simulation with PpVα peptide analogs; (**B**) RMSD value of PpVα peptide analogs during molecular dynamics simulation with Na_v_ channel receptor; (**C**) The Rg of PpVα peptide analogs with the Na_v_ channel receptor; (**D**) The hydrogen bonds that were generated between the Na_v_ channel receptor and the peptides.

**Figure 5:**
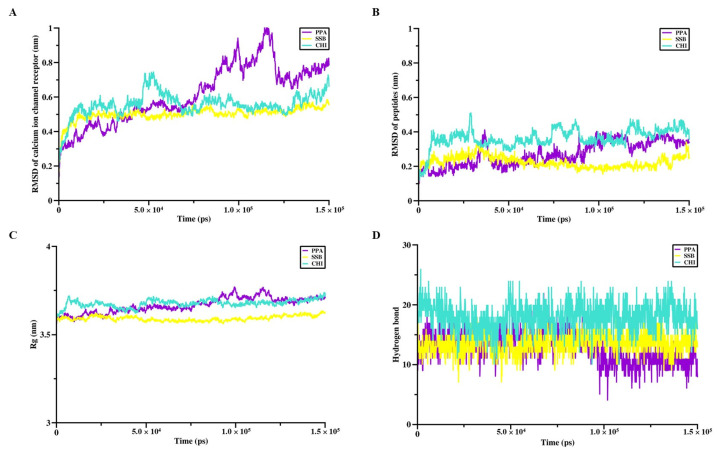
Molecular dynamics simulation between the Ca^2+^-channel and the PpVα peptide analogs. (**A**) RMSD value of Ca_v_ channel receptor during molecular dynamics simulation with PpVα peptide analogs; (**B**) RMSD value of PpVα peptide analogs during molecular dynamics simulation with Ca_v_ channel receptor; (**C**) The Rg of PpVα peptide analogs with the Ca_v_ channel receptor; (**D**) The hydrogen bonds that were generated between the Ca_v_ channel receptor and the peptides.

After molecular docking, the peptide complexes with the Nav channel receptor and the Cav channel receptor were subjected to a 150-ns GROMACS molecular dynamics simulation. As shown in Figure 4A, the RMSD value of the Na_v_ channel receptor complexed with SSB reached a lower plateau than PPA and CHI. When comparing the RMSD values of PPA, SSB, and CHI without the Na_v_ channel receptor, it was found that SSB maintained the lowest RMSD, indicating the highest stability, followed by CHI, with PPA exhibiting the highest RMSD, thus the lowest stability (Figure 4B). Regarding the radius of gyration (Rg), all three peptides showed similar values (Figure 4C). Additionally, SSB formed more hydrogen bonds with the Na_v_ channel receptor compared to PPA and CHI (Figure 4D). Similar to what was observed with the Na_v_ channel, SSB demonstrated superior performance in terms of the receptor’s RMSD values (Figure 5A), the RMSD without the receptor, and Rg in the binding process with the calcium ion channel receptor, exhibiting higher stability than CHI and PPA (Figure 5B,C). The number of hydrogen bonds formed between SSB and the Ca_v_ channel receptor was only slightly less than that of CHI but more than PPA (Figure 5D).

In comparison, CHI outperformed PPA in terms of the receptor’s RMSD values and the number of hydrogen bonds, while their performance in RMSD without the receptor and Rg was comparable. Overall, SSB showed the best binding stability with both the sodium ion channel receptor and the calcium ion channel receptor. In contrast, PPA and CHI performed similarly, with CHI slightly outperforming PPA.

Each peptide–ion channel complex was solvated in a cubic box using the TIP3P water model, ensuring a minimum distance of 1.0 nm between the protein surface and the box edge. The systems were neutralized by adding the appropriate number of counterions, followed by the addition of Na^+^ and Cl^−^ ions to reach a final ionic concentration of 0.15 M, mimicking physiological conditions. Energy minimization was performed prior to equilibration and production runs, and molecular dynamics simulations were conducted for 150 ns using the GROMACS software.

### 2.4. Sodium Currents I_Na_ Are Affected by PpVα Peptide Analogs in Bovine Chromaffin Cells

**Figure 6:**
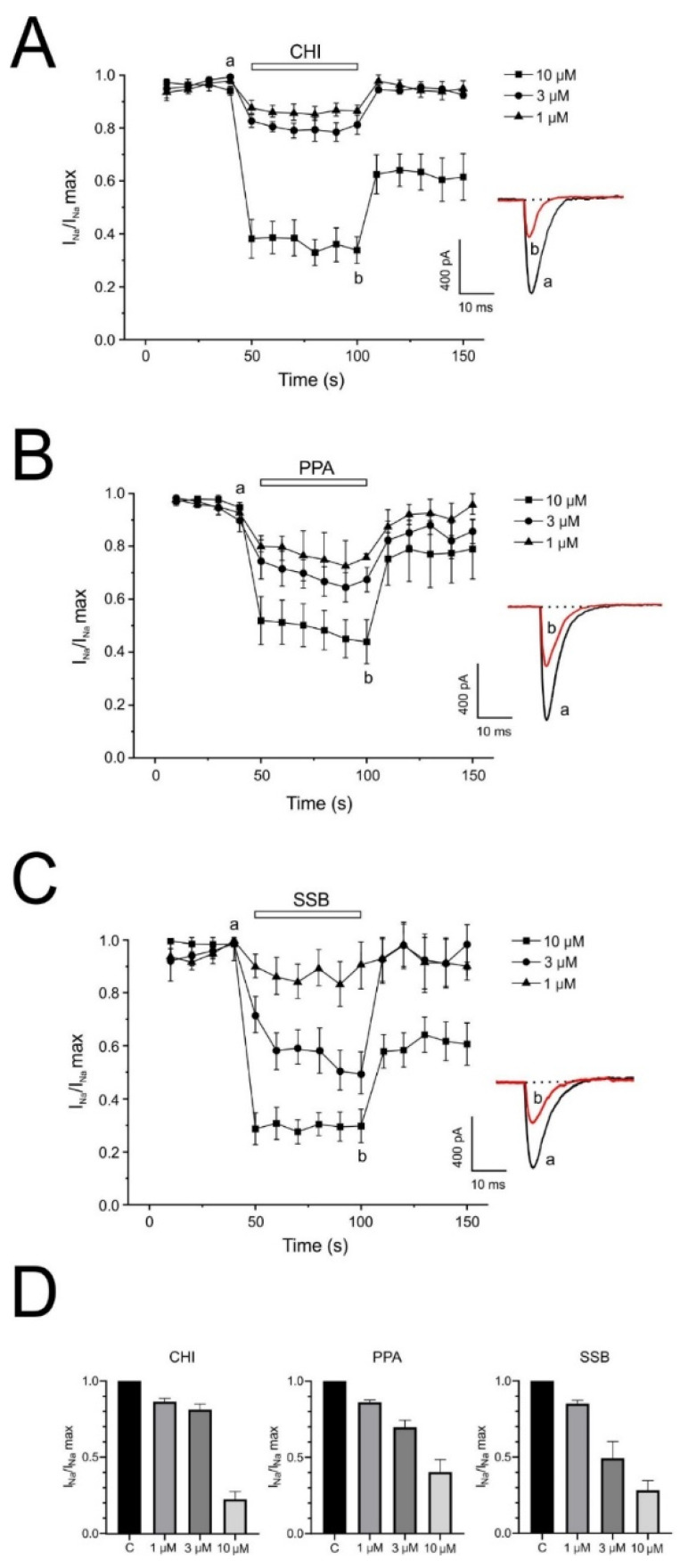
Reduction of sodium inward current (I_Na_) caused by PpVα peptide analogs in bovine chromaffin cells. The average time course of I_Na_ in control conditions and after perfusion with varying concentrations of each PpVα peptide analog is shown at the right. CHI (**A**), PPA (**B**) and SBB (**C**) in 0 mM Ca^2+^ external solution. Inset Original traces obtained in the control condition (a) and at the end of 10 μM PpVα peptide analogs application (b). (**D**) Average results of the % of current inhibited (ordinate scale) after 1 min perfusion with each concentration of PpVα peptide analogs (abscissa scale). A separate bovine chromaffin cell was used for each concentration of PpVα peptide analog. The graph plots peak current data, normalized to the mean value of the control period, and expresses them as the mean ± SEM of 4–6 experiments.

In the excitatory cell types, Na^+^ is the ion responsible for depolarization. In the experiments shown in Figure 6, each bovine chromaffin cell was individually voltage-clamped and stimulated with 10 ms depolarizing pulses to 0 mV, applied at 20 s intervals from a holding potential of −80 mV. The inward I_Na_ currents were elicited with 0 mM extracellular Ca^2+^. In 8 cells tested, the average current amounted to 598 ± 91 pA. This current showed no appreciable decline during the approximately 10 min testing period; if a decrease was observed, the cell was discarded. Once the initial current was stabilized, each cell was superfused with a single concentration of each PpVα peptide analog until the effect stabilized for 3 min—the partial recovery after washout precluded making a cumulative concentration-dependent response in the same cell. The current was normalized once I**_N_**_a_ had stabilized at the beginning of each recording (I**_N_**_a_/I**_N_**_a_ max). Figure 6 shows the averaged time courses of I**_N_**_a_ inhibition by three concentrations for every PpVα peptide analogs (1, 3, and 10 µM): (Figure 6A) CHI, (Figure 6B) PPA, and (Figure 6C) SSB.

At the low concentrations used (1 and 3 μM), CHI had no effect on I_Na_. The block was significant at 10 μM (1 μM, 14.79 ± 2.16%; 3 μM, 22.05 ± 3.61% and 10 μM, 75.57 ± 5.05%; n = 4–6 cells) (Figure 6A). The blocking effect exerted by PPA was (1 μM, 14.80 ± 5.05%; 3 μM, 28.92 ± 4.60% and 10 μM, 54.32 ± 8.29%; n = 3–5 cells) (Figure 6B). And block exerted by SBB was (1 μM, 87.34 ± 8.06%; 3 μM, 52.01 ± 11.02% and 10 μM, 73.76 ± 6.37%; n = 4–5 cells) (Figure 6C). The higher concentration of PpVα peptide analogs (10 μM) evoked a more significant block, achieving 80% after 10 s of peptide application. Insets show representative traces from control conditions and from 3 min after perfusion with peptide analogs (10 μM). Figure 6D shows the concentration-dependent response data for the blocking effect of the peptide analog indicated on peak I_Na_. Inhibition of I_Na_ was individually measured in each cell at the end of the 3-min superfusion period for each concentration of PpVα peptide analog. The half-maximal inhibitory concentration (IC_50_) values for sodium current blockade by CHI, PPA, and SSB were determined to be 7.65, 9.08, and 3.15 μM, respectively. These values were obtained from fitting the experimental data to an exponential function.

### 2.5. PpVα Peptide Analogs Do Not Affect Potassium Currents in Bovine Chromaffin Cells

**Figure 7:**
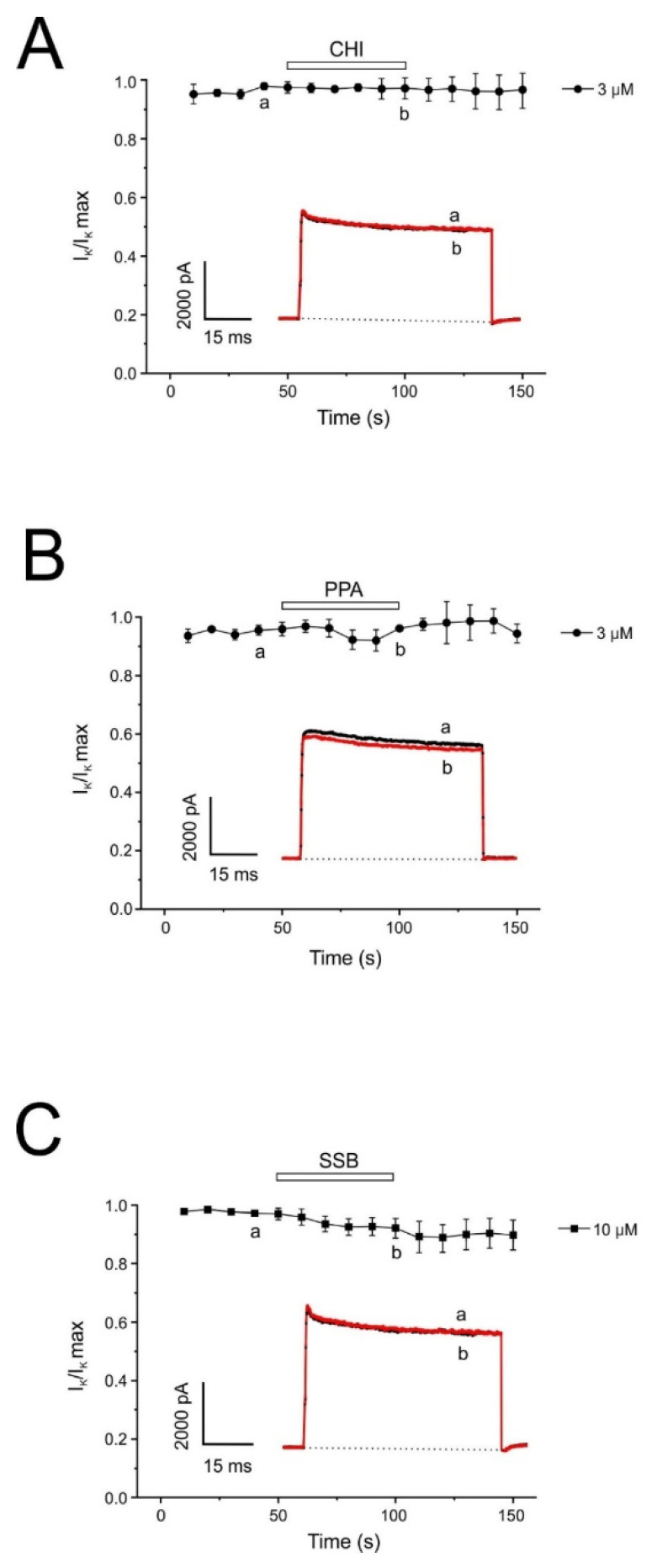
Testing inhibition of voltage-dependent K^+^ currents (I_Kv_)by PpVα peptide analogs. Average time courses of I_Kv_ obtained with the protocol indicated during and after 3 min perfusion of the concentrations of PpVα peptide analogs shown at the right and applied during the time indicated by the top horizontal bar (3 min) CHI (**A**), PPA (**B**) and SBB (**C**). Inset: Original traces were obtained in the control condition (a) and at the end of the 10 mM PpVα peptide analog application (b). Average results of the % of current inhibited (ordinate scale) after 1 min perfusion with each PpVα peptide analog concentration (abscissa scale). The graphs plot data normalized to the control period mean and expressed as the mean ± SEM of 3–4 experiments.

As in most cell types, K^+^ is the ion responsible for repolarization in the bovine chromaffin cell. This set of experiments studied the effects of PpVα peptide analogs on the voltage-dependent potassium current (I_Kv_). Figure 7 shows the time course of IKv elicited by 400 ms depolarizing pulses to 100 mV from a holding potential of −80 mV, delivered at 20 s intervals. The I_Kv_ remained stable during the recording period and averaged 2585 ± 138 pA (n = 7). Once the initial current stabilized, each cell was superfused with a single concentration of PpVα peptide analogs ) until the effect stabilized after 3 min. The PpVα peptide analog subtypes tested did not exert any appreciable effect on voltage-dependent potassium current even at high concentration (10 µM), showing a block of 1.21 ± 2.42% (CHI) (Figure 7A); 14.04 ± 5.99% (PPA) (Figure 7B); 12.84 ± 4.19% (SSB) (Figure 7C) (n = 3–4 cells). Even at the highest concentration of SSB tested (10 µM), no effect on I_Kv_ was observed. The current was normalized from each recording (IKv/IKv max) at the beginning of the recording. The inset shows original traces corresponding to control conditions (a) and 1 min after perfusion with 10 μM of each PpVα peptide analog (b).

### 2.6. Time-Course Block of Voltage-Dependent Ca^2+^ Channel Exerted by PpVα Peptide Analogs

**Figure 8:**
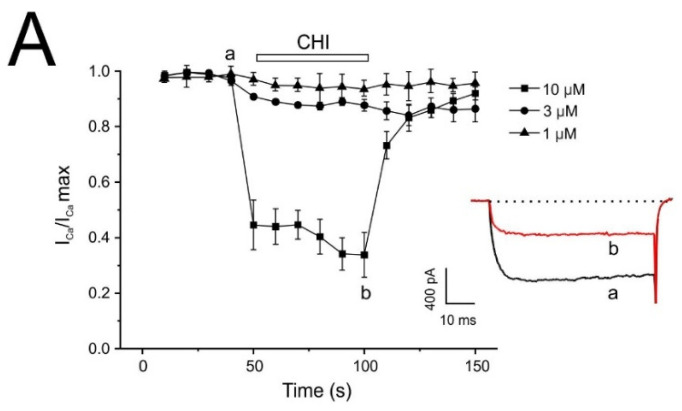

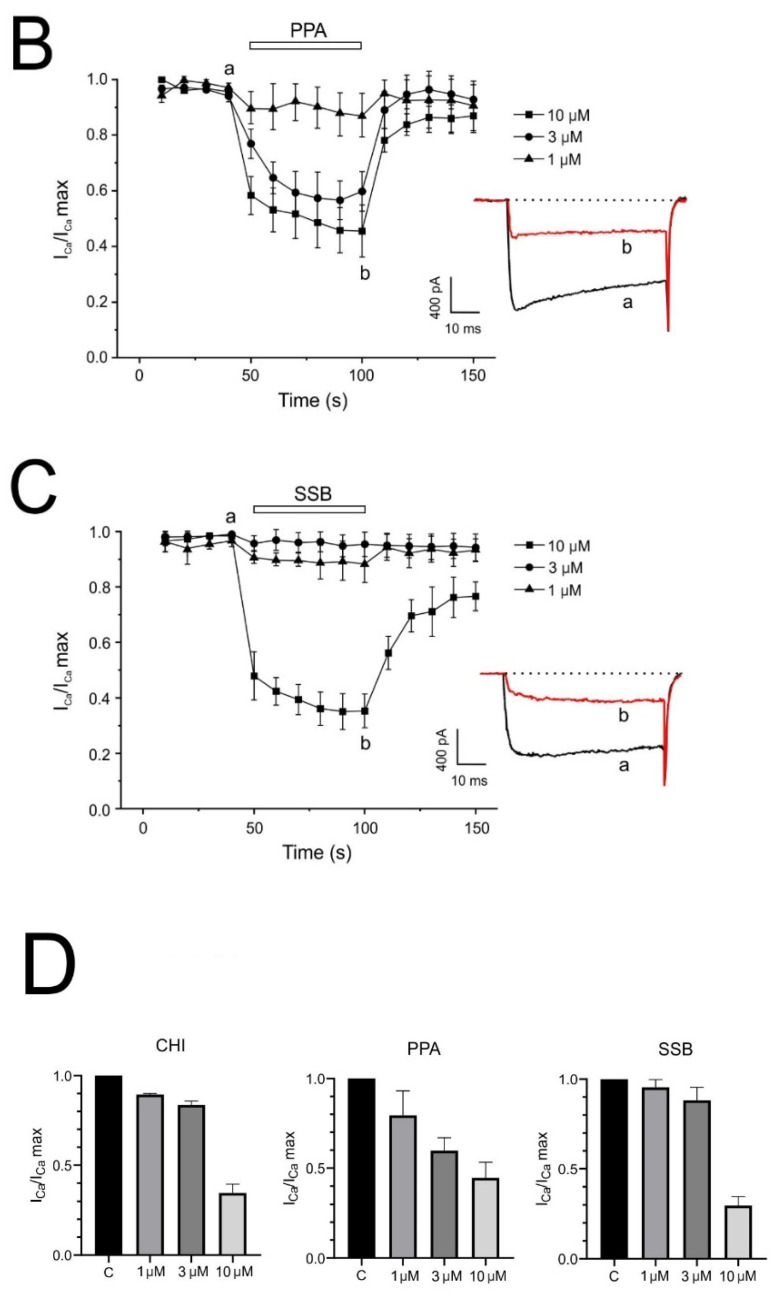
Block of voltage-dependent Ca^2+^-channel in bovine chromaffin cells by PpVα peptide analogs—averaged time-course inhibition study of the whole-cell inward Ca^2+^ current by PpVα peptide analogs (**A**–**C**). 10 mM Ca^2+^ and Tetrodotoxin (5 μM) were added to the external solution to measure I_Ca_. Data points represent the maximum peak current initially obtained. The top horizontal bars indicate the superfusion of the concentrations of PpVα peptide analogs shown at the right: (**A**), CHI; (**B**), PPA; (**C**), SSB. A separate cell was used for each concentration of PpVα peptide analog. Insets. Original traces were obtained in the control condition (a) and at the end of the PpVα peptide analogs application, 10 mM (b). (**D**) Averaged results of the % of current inhibited (ordinate scale) after 1 min superfusion with each PpVα peptide analog type concentration, abscissa scale. The graphs plot data normalized to the control period mean and expressed as the mean ± SEM of 5–6 experiments.

In the experiments shown in Figure 8, each voltage-clamped cell was stimulated with 50 ms depolarizing pulses to 0 mV, applied at 20 s intervals from a holding potential of −80 mV. The inward I_Ca_ currents were elicited with 10 mM extracellular Ca^2+^. In 9 cells tested, the average current was 634 ± 59 pA. This current showed no appreciable decline during the approximately 10 min testing period; if a decrease was observed, the cell was discarded. Once the initial current stabilized, each cell was superfused with a single PpVα peptide analog concentration until the effect stabilized for 3 min. As before, the partial recovery of the current after washout precluded the analysis of cumulative concentration-response in the same cell. The current was normalized once I_Ca_ stabilized at the beginning of the recording (I_Ca_/I_Ca_ max). Figure 8A–C shows the average time courses of I_Ca_ inhibition by three concentrations (1, 3, and 10 µM) of each PpVα peptide analog.

At a low concentration (1 μM), PpVα peptide analogs produce a slight block of I_Ca_, but without being significant for any of them (10.74 ± 0.61% for CHI, 19.92 ± 5.32% for PPA, and 10.10 ± 7.22% for SSB, n = 3–5 cells). The blocking effects exerted by CHI and PPA showed concentration dependence (14.28 ± 2.08% and 38.50 ± 7.14% at 3 μM; 63.43 ± 4.76% and 52.86 ± 8.69% at 10 μM) (Figure 8A and Figure 8B, respectively). The highest concentration of SSB used (10 μM) also evoked a significant block of I_Ca,_ 74.46 ± 5.00% (Figure 8C). Insets show representative traces from control conditions and 1 min after perfusion with the corresponding PpVα peptide analog (10 μM). Figure 8D shows the average block on peak I_Ca_ exerted by the different concentrations of the PpVα peptide analog used. ICa inhibition was measured in each cell individually at the end of the 1-min superfusion period for each concentration of PpVα peptide analog. The half-maximal inhibitory concentration (IC_50_) values for calcium current blockade by CHI, PPA, and SSB were determined to be 8.88, 8.77, and 9.68 μM, respectively. These values were obtained from fitting the experimental data to an exponential function.

### 2.7. PpVα Peptide Analogs Reduce the Intracellular Calcium Level in Chromaffin Cells

**Figure 9:**
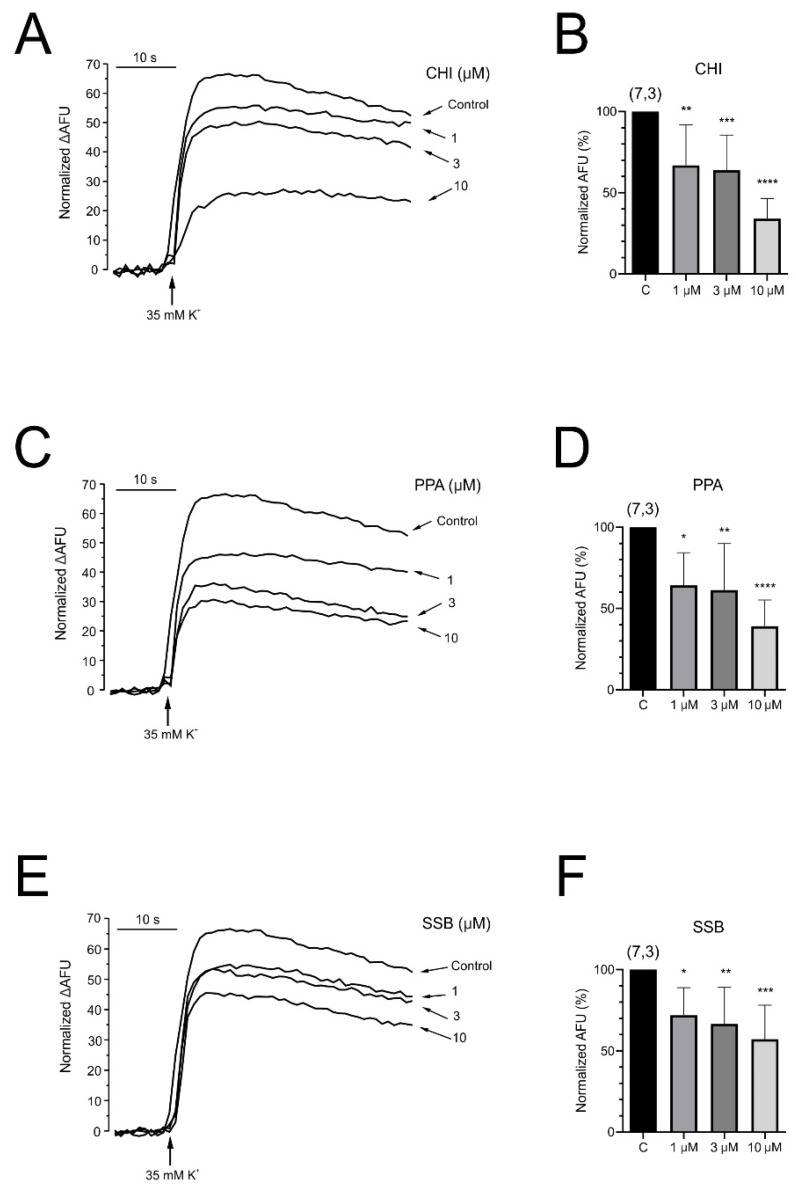
Effects of PpVα peptide analogs on intracellular calcium level in bovine chromaffin cells. Original traces of the Fluo-4 fluorescence induced by 35 mM K^+^ in the absence (control) or presence of CHI (**A**), PPA (**C**), or SSB (**E**) at the indicated concentrations. Bar graphs of normalized fluorescence compare the effect of fluorescence in the absence (control) or in the presence of CHI (**B**), PPA (**D**), or SSB (**F**) at the concentrations indicated on the abscissa. Control data means the Fluo-4 fluorescence induced by K^+^ without peptides. The values were normalized to the maximum fluorescence intensity produced with K^+^ (35 mM). The data are presented as mean ± SEM. AFU, arbitrary fluorescence units. Results were considered statistically significant when * *p* ≤ 0.05, ** *p* ≤ 0.01, or *** *p* ≤ 0.001, **** *p* < 0.0001.

Previous experiments have shown a drastic reduction in calcium currents induced by PpVα peptide analogs. We wondered whether this effect may be related to alterations in cellular calcium homeostasis, which are involved in cellular excitability and neurotransmitter secretion. To investigate whether the calcium necessary to activate the neurotransmitter release may be affected, we have designed the experiments represented in Figure 9. The measurements of the changes in intracellular calcium level ([Ca^2+^]_c_) in populations were carried out by using the fluorescent probe Fluo-4 AM. After 10 s, a stimulus with K^+^ (35 µM) was applied, producing a rise in fluorescence that was maintained for the remainder of the experiment, which lasted for a minute. Panels A, C, and E show original recordings obtained in the absence (control) and the presence of PpVα peptide analogs (1, 3, and 10 μM). Applying PpVα peptide analogs without cellular stimulation showed no fluorescence signal enhancement. Panels B, D, and F show the original values of the [Ca^2+^]_c_ reduction caused by increasing concentrations of the three PpVα peptide analogs. The reduction of [Ca^2+^]_c_ exerted by CHI, PPA, and SSB showed a concentration-dependent effect. Thus, each PpVα peptide analog type concentration used led to a reduction in intracellular calcium levels of 33.2 ± 9.4, 38.5 ± 7.1, and 28.1 ± 6.0% for 1 μM; 36.0 ± 8.2, 38.7 ± 10.8, and 33.3 ± 7.9% for 3 μM and 65.0 ± 4.7, 60.9 ± 6.1 and 42.7 ± 7.4% for 10 μM, respectively. CHI and PPA were more effective than SSB at 10 μM, reducing fluorescence by more than 60% (*p* ≤ 0.001) compared with baseline. This effect on the calcium-dependent fluorescence signal becomes statistically significant across all concentrations of PpVα peptide analogs tested (see figure legend). The IC_50_ values for depression of the intracellular calcium increment by CHI, PPA, and SSB were 6.89, 6.95, and 26.03 μM, respectively. These values were obtained from fitting the experimental data to an exponential function.

### 2.8. Neuroprotective Effects of PpVα Peptide Analogs Observed in Human Neuroblastoma Cells

Chromaffin cells are most commonly used in studies of exocytosis and the release of neurotransmitters. In contrast, neuroblastoma lines (SH-SY5Y) tend to be more robust in culture and better withstand stressful experimental conditions, such as exposure to toxic agents or antioxidants in experiments of cytoprotection.

**Figure 10:**
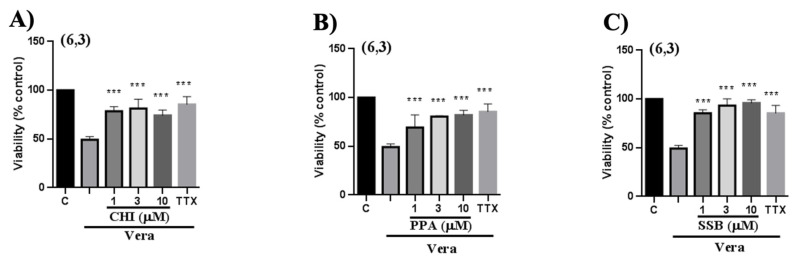
The neuroprotective effect of PpVα peptide analogs in reversing the neurotoxicity caused by veratridine in neuroblastoma cells. Experimental protocol consisting of two pre-incubation periods of 24 h with the peptides at concentrations of 1 μM, 3 μM and 10 μM, followed by a co-incubation period of 24 h with veratridine and CHI (**A**), PPA (**B**), or SSB (**C**). Statistical differences in neuronal damage caused by veratridine were determined using a one-way analysis of variance (ANOVA). *** *p* ≤ 0.001.

The neuroprotective properties of the PPA, SSB, and CHI peptides were evaluated in human neuroblastoma cells using veratridine as a neuroinflammation inducer. Cells pre- and co-incubated with veratridine for 24 h had a 50% mortality rate compared with untreated cells under the same culture conditions. Treatment of cells with the peptide analogs added after veratridine resulted in a significant reduction in cell mortality, with cell viability restored to 80–100% (*p* < 0.001) depending on the peptide concentration (Figure 10; Table 2). PPA showed lower neuroprotection than SSB and CHI. In the same assay, tetrodotoxin almost wholly reverted the cellular damage, as expected for a veratridine antagonist. Overall, all three peptides demonstrated a significant cytoprotective effect (*p* < 0.005), counteracting veratridine’s cytotoxicity and restoring cell viability.

**Figure 11:**
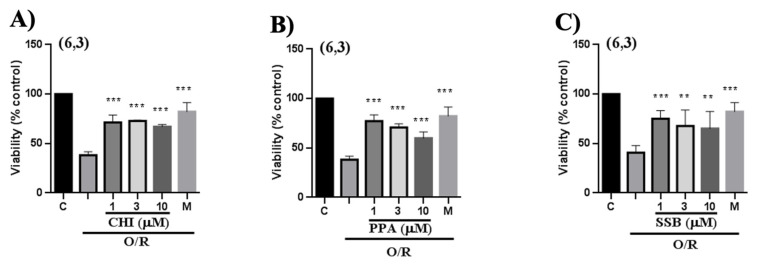
The neuroprotective effect of PpVα peptide analogs in reversing the oxidative stress caused by oligomycin and rotenone in neuroblastoma cells. Experimental protocol consisting of two pre-incubation periods of 24 h with the peptides CHI (**A**), PPA (**B**), and SSB (**C**) at concentrations 1, 3, and 10 μM, followed by a co-incubation period of 24 h with the same peptides and veratridine. Statistical differences in the neuronal damage caused by O/R and the lactate dehydrogenase assay were determined. Data was tested with one-way analysis of variance (ANOVA), and results were considered statistically significant when *p*≤ 0.01 **, *p* ≤ 0.001 ***.

Additionally, we evaluated whether the PpVα peptide analogs may revert mitochondrial oxidative stress. The reversal of the harmful effect of endogenous reactive oxygen species on cell viability in neuroblastoma cells mediated by single PpVα peptide analogs was evaluated following exposure of SH-SY5Y cells to a mixture of oligomycin and rotenone (O/R) for 24 h and subsequent treatment with the single PpVα peptide analogs. Oligomycin inhibits ATP synthase, interfering with ATP production in the mitochondria, and rotenone inhibits complex I in the mitochondrial respiratory chain. After treatment, a substantial reversal of cell damage occurred. The combination of oligomycin and rotenone significantly induced cellular damage, evidenced by a 40% decrease in cell viability as measured by the lactate dehydrogenase assay. Remarkably, when cells were pre-treated with the O/R (3 μM/4 μM) mixture for 24 h and then exposed to the PpVα peptide analogs, an impressive 70% reversion in cell damage across all three peptides tested (Figure 11). In comparison with the positive control melatonin—a naturally occurring antioxidant in which the reversion of cell damage reached approximately 80%, the three PpVα peptide analogs exhibited comparable efficiency to circumvent cell injury and death. These findings highlight the therapeutic potential of peptide analogs in mitigating cellular damage caused by reactive oxygen species, suggesting promising avenues for further investigation in neuroprotective strategies. Table 3 summarizes the observed quantitative effects for each treatment condition.

## 3. Discussion

One of the neurotoxic components of the sea trumpet polyp Protopalythoa variabilis (Cnidaria, Anthozoa) is a 26-residue, V-shape helical peptide (PpVα). This study investigated the synthetic versions of this peptide (PPA), the single disulfide bond, folded analog (SSB), and the chimeric peptide (CHI) (Table 1) for their activity on ion channels responsible for cellular excitability and synaptic transmission. The molecular docking and MD simulation data indicated the interaction of PpVα peptide analogs with the critical residues at the binding sites of the human voltage-gated sodium (Nav1.7) channel and human N-type voltage-gated calcium (Ca_V2_._2_) channel. Thus, we analyzed the acute effects of these three different PpVα peptide analogs on the excitability of isolated bovine chromaffin cells. We explored their impact on ionic currents for sodium, potassium, and calcium, as well as on the generation and propagation of intracellular calcium. Thus, PpVα peptide analogs (Table 1) produced (1) a drastic and reversible block of the voltage-dependent Na^+^ currents, (2) a gradual and reversible block of voltage-dependent Ca^2+^ currents, (3) no effect on the voltage-gated K^+^ conductance even at high concentrations, (4) a pronounced blocking effect on the intracellular Ca^2+^ signal. These effects were more relevant for the single disulfide-bonded PpVα analog (SSB) and the chimeric peptide (CHI). Moreover, the PpVα peptide analogs showed in vitro cytoprotective effects, reversing injury caused by veratridine, rotenone, and oligomycin by dysregulating compounds that dysregulate ion channels and inducing mitochondrial oxidative stress.

Marine organisms are considered valuable sources of bioactive compounds for biomedical research and pharmaceutical development [27]. Among these marine natural chemicals, peptides—especially those produced through non-ribosomal peptide biosynthesis are molecules of interest [28,29]. Additionally, gene-encoded neurotoxic peptides from venomous marine organisms, such as mollusks (e.g., sea snails of the genus Conus) and cnidarians, constitute a structurally diverse collection of class compounds, displaying high specificity and selectivity—critical properties for discriminating pharmacological targets and developing therapeutic molecules [30,31]. Cnidarians, which encompass thousands of marine species distributed across six main classes, are mostly toxigenic and include anemones, jellyfish, and corals, and are valuable biological reservoirs that contain numerous neurotoxic peptides and pore-forming toxins, among other classes of peptide toxins [11,18]. Classical pharmacological studies, as well as recent omics techniques have uncovered target-specific neurotoxins expressed in cnidarians that can serve as probes for testing a diversity of ion channels [16,17,32,33]. Currently, most neurotoxic peptides found in cnidarians, particularly sea anemones and jellyfish, affect subtypes of voltage-gate.d sodium and potassium ion channels. Less characterized are cnidarian toxins that act on other types of ion channels, such as ligand-gated ion channels. Previously, Liao and colleagues [14] reported the well-characterized cnidarian neurotoxic peptides that modulate ion channels and can serve as analgesic (anti-pain), anti-epileptic, and neuroprotective agents. These neurotoxic peptides comprise inhibitors of potassium and sodium channels, acid-sensing ion channels, and TRPV1, as well as Kunitz-type inhibitors with well-defined structures.

A short peptide with a V-shape helical configuration was found in the transcriptome of the anthozoan *P. variabilis* that shared high similarity with its counterpart from the sea mat coral *Palythoa* spp. [17]. Functional analysis of synthetic peptide analogs of this V-shape helical peptide (PpVα) and its analogs indicated that the folded peptide interacts virtually with the voltage-dependent sodium ion channel (Na_V_1.7 subtype). In vivo, the folded peptide was less toxic than the linear peptide in the zebrafish model. Additionally, it was more effective than the linear unfolded peptide in suppressing 6-OHDA-induced neurotoxicity on locomotor behavior in zebrafish. Notably, folded-PpVα prevented the 6-OHDA-induced excessive ROS generation and subsequent dopaminergic neuron loss [14]. These findings, using the PTZ-induced epileptic model in zebrafish larvae, were significant, demonstrating the neuroprotective and anti-epilepsy activities of PpVα peptides.

The interaction sites and binding affinities between PpVα peptide analogs and ion channels were analyzed using molecular docking and MD simulations. All in silico-constructed peptide models, along with their structural characteristics (Table 3), were stable, as indicated by the calculated RMSD values (Figure 4A–D and Figure 5A–D). The tarantula protoxin-II (ProTx2) and the *Conus* ω-conotoxin MVIIA-derived ziconotide are potent and selective antagonists of Nav1.7 and Ca_v_2.2. ion channels, respectively, served as references for mapping interaction sites and for comparing the binding affinities of PpVα peptide analogs in peptide-ion channel complexes. The peptides PPA, SSB, and CHI showed similar affinities for the Na^+^-channel, which was slightly less potent than tarantula ProTx2. The interaction and affinity of PPA, SSB, and CHI with Ca_v_2.2 ion channel were equivalent but quantitatively inferior to the conotoxin-derived ziconotide. Notably, the amino acid residues in the ion channels that PPA and SSB peptides can interact with are coincident with the critical residues in the binding sites of Na_v_ inhibitors and Ca_v_2.2. which ziconotide also binds. Combining molecular docking and MD simulation corroborated the experimental data at the atomic level, was consistent with the subsequent electrophysiological measurements, and supported the finding that the Na^+^-channel is the preferred target for PpVα peptide analogs.

The marked difference between these PpVα peptide analogs is the presence of a preformed disulfide bridge in SSB, which appears to confer greater efficacy and better topological accommodation within the ion channel. Disulfide bridges are commonly present in peptide toxins and have pharmacological advantages for peptide stability, functionality, and bioavailability [34,35,36]. Compared with sodium-ion channels, and despite being less effective, the peptide analogs studied here still displayed a nearly 50% block of calcium relative to baseline, along with a corresponding lower affinity for the calcium channel, as determined by molecular docking. Although there are 9 subtypes of Na+ channels, bovine chromaffin cells express only 2: Nav1.3 and Nav1.7. Due to its higher concentration in neuroendocrine cells, the Nav1.7 subtype is considered the most plausible target for the actions of peptides and other substances in voltage-clamp studies and in cytoprotection. On the other hand, Nav1.3 is present in peripheral neurons, and bovine chromaffin cells do not possess more than one subtype. Therefore, sodium channel inactivation in bovine chromaffin cells may occur primarily via Nav1.7.

It is interesting to note that the crude venom of the zoanthid (cnidarian) *Palythoa caribaeorum*, from which the homologous PpVα peptide was initially characterized, caused inhibition of K^+^ and Ca^2+^ channels in the superior cervical ganglion neurons of the rat, but not Na^+^-channels [16]. The isolated fraction containing the native PpVα homolog peptide caused delayed sodium current inactivation. In contrast, the purified PpVα homolog interfered with the A-type transient outward and delayed rectifier subtypes of KV channels in cultured rat sympathetic neurons [18]. These apparent discrepancies in the peptide’s influence and selectivity across different ion channel types stem from the use of crude venom and its fractions, as well as the experimental models employed. Our data indicated that the synthetic PpVα peptide analogs were preferentially selective for Na_v_ and Ca_V_ channels in bovine chromaffin cells. Regarding Na_V_ ion channels, our present work reinforces the findings of previous work by Liao and colleagues [14] on the virtual interaction of the folded-PpVα peptide with the human neuronal Nav 1.7 channel. Thus, to our knowledge, this is the first time that individual PpVα peptide analogs have been studied for their ability to modulate Na^+^ and Ca^2+^ channels and their respective ion currents in a cellular model using the patch-clamp technique.

The adrenal medulla is predominantly composed of chromaffin cells, which, as sympathetic neurons, develop from the neural crest. Chromaffin cells are modified postganglionic sympathetic neurons. They are excitable cells with neuron-like electrical properties [37] with the capacity to synthesize, store, and release adrenaline and noradrenaline (for review, see [38]). They are among the most widely used cellular models for investigating the molecular mechanisms underlying cellular excitability and neurotransmitter release [39]. In most excitable cells, the input current that triggers the action potential is produced by activating voltage-dependent Na^+^ channels [40]. We demonstrate here that PpVα peptide analogs, can block Na^+^ current in chromaffin cells in vitro. These effects on TTX-sensitive Na^+^ channels occur in the low micromolar range, suggesting that physiologically relevant concentrations of PpVα peptide analogs may regulate the activity of neurons expressing Na+ channels in the brain.

Additionally, K^+^ channels play a critical role in repolarizing the action potential, setting the resting potential, modifying cellular excitability, and regulating the temporal pattern of action potential firing [41,42]. Among the diverse K^+^ channels, the K^+^ current in chromaffin cells is voltage-dependent. Our results show that chromaffin cells exhibit little or no inhibition of voltage-dependent IK by PpVα peptide analogs.

Ca^2+^ ions play an essential role in neurotransmitter release [43] due to their influx through the voltage-dependent calcium channels, essentially through N- and P-types in neurons [44] and L-type in neuroendocrine cells [45]. The inward Ca^2+^ current in bovine chromaffin cells amounted to 15% for L, 80% for N- and P/Q-type, and 15% for R-type channels [46]. We show that PpV (peptide analogs) block calcium currents in a time- and dose-dependent manner, and that this block was partially reversible. The highest concentration used (10 µM) suppressed calcium influx by 80%; therefore, all Ca^2+^ channel subtypes present in chromaffin cells appeared to be affected. The lack of effect on Ca^2+^ current kinetics suggests that the block occurs regardless of the channel’s open or closed state. This finding, linked to PpVα peptide analogs’ selectivity towards neuronal tissue, may have essential neurotoxic relevance, as L channels are involved in neuronal induction of gene expression. In contrast, N and P/Q channels are involved in neurotransmission [47]. Additionally, it has been reported that during action potential firing, calcium currents are involved in both the early, slowly activating phase (pre-spike) carried by L-type channel that contributes to the pacemaker potential and the rapid action potential upstroke, and in the late, short-lasting component (post spike) carried by non-L-type channels that sustains the AP repolarization [48].

It is essential to note that although the present work focuses on a general assessment of the neurotoxic effects of these toxins, future studies should analyze the specificity of these neurotoxic peptides for the different Cav calcium channel subtypes L, N, P/Q, and R. The Ca_v_ calcium channel subtypes (L, N, P/Q, and R) have critical functional relevance in cellular physiology, especially in the nervous system and excitable tissues. Each of these subtypes is involved in key cellular processes, including neuronal excitability, neurotransmitter release, muscle contraction, and gene regulation. Evaluating the specificity of neurotoxins for each subtype is crucial to understanding their therapeutic potential or their pathological effects (for details, see [49,50,51]).

The effect of exposure of chromaffin cells to PpVα peptide analogs on the intracellular fluctuation of calcium ions was evaluated with a fluorescent probe (Fluo-4 acetoxymethyl ester). The significant decrease in intracellular calcium concentration caused in such a neuronal cell by PpVα peptide analogs was voltage- and concentration-dependent (CHI > PPA > SSB). Such an effect is noteworthy, as intracellular calcium levels and calcium homeostasis are involved innumerous biological processes, including neuronal excitability and neurotransmitter secretion, as well as neuroinflammation and neuronal death [52]. Thus far, in concert with Na^+^ and Ca^2+^ channel blockage, the voltage- and concentration-dependent decrease in intracellular calcium flow could interrupt neurotransmitter release and contribute to the immobilization of prey and victims. In fact, an in vivo toxicity test with zebrafish larvae exposed to different concentrations of the PpVα peptide (PPA) (1–30 µM) at three different times showed a decrease in survival rate to a minimum, for example, within 20 min at 3 µM peptide. The calculated concentration of peptides that caused 50% of death (LD_50_) in zebrafish larvae was 21.23 µM for the linear PpVα peptide (PPA) and 10.88 µM for the folded PpVα (SSB) [14,53].

Since the PpVα peptide analogs inhibited sodium and calcium ion channels in bovine chromaffin cells, interfering with intracellular calcium ion levels, we investigated the presumed neuroprotective activity in human neuroblastoma (SH-SY5Y) cells. Chromaffin cells are most commonly used in studies of exocytosis and catecholamine release, which may not be central to all cytoprotection research. Neuroblastoma lines tend to be more robust in culture than chromaffin cells and better withstand stressful experimental conditions, such as the use of toxic agents or antioxidants in cytoprotection experiments. Neuroblastoma cells are ideal for studying apoptosis, autophagy, and oxidative stress, which are critical pathways in cytoprotection. Furthermore, another reason for the extensive use of human neuroblastoma cells, although not the focus of this study, is that they are easier to genetically manipulate, allowing for the study of specific pathways involved in cytoprotection. The cell viability assay with MTT was performed after 24 h of veratridine-induced stress in SH-SY5Y cells, followed by treatment with PpVα peptides (CHI, SSB, and PPA) and TTX as a positive control. Veratridine is a neurotoxic alkaloid that induces sensitization and cell death in various cell types, including bovine chromaffin cells [54]. This toxic compound prevents sodium channel inactivation and increases the influx of sodium ions [55]. In this essay, the analogs of the PpVα peptide exhibited a cytoprotective effect similar to the positive control, TTX (a specific blocker of voltage-gated sodium channels). These results recapitulate that the peptides block the sodium channel, as TTX does, impairing sodium influx and protecting SH-SY5Y cells from veratridine-induced damage. In the other neuroprotective assay with SH-SY5Y cells, cells were exposed to a combination of the organic toxins oligomycin and rotenone (O/R) to inhibit mitochondrial respiratory chain phosphorylation, disrupting cellular metabolism. In this essay, oligomycin inhibits ATP synthase, thereby interfering with mitochondrial ATP production, and rotenone inhibits complex I in the mitochondrial respiratory chain. Both toxins act on the mitochondria, disrupting ATP production, which increases the production of reactive oxygen species ROS that can lead to oxidative stress and result in cellular damage. Interestingly, exposure of neuroblastoma cells pre-treated with O/R to all single PpVα peptide analogs reversed the damage caused by the mitochondrial disruptors and stressors. The cyto- and neuroprotective effects agree with previous data by Liao and collaborators [14,53] using rat adrenal pheochromocytoma (PC12) cells. The production of reactive species leads to oxidative stress, which can disrupt ion channel function and contribute to symptoms of neurodegenerative disease. Reactive oxygen species, by oxidizing cysteine residues, alter the function of ion channels, leading to an imbalance in ion homeostasis.

Although PpVα and its analogs are relatively large peptides (~26 residues), our data indicate that their primary effects on mitochondrial function and oxidative stress are likely indirect. Electrophysiological, molecular docking, and molecular dynamics data indicate that PpVα analogs selectively block voltage-gated Na^+^ (NaV1.7) and Ca^2+^ (CaV2.2) channels at the plasma membrane, leading to a significant reduction in intracellular calcium levels. Because mitochondrial function is highly sensitive to calcium homeostasis, modulation of ion fluxes at the plasma membrane can indirectly influence mitochondrial oxidative stress without requiring direct peptide entry into mitochondria.

While peptide internalization into the cytosol via endocytosis or other mechanisms cannot be completely ruled out, the evidence from our cytoprotection assays in SH-SY5Y cells treated with mitochondrial toxins (oligomycin and rotenone) supports the view that the neuroprotective and anti-oxidative effects are mediated primarily through ion channel modulation rather than direct mitochondrial targeting.

In summary, we have evaluated the effects of acute administration of PpVα peptide analogs on ionic currents involved in neurotransmitter release and cellular excitability. Thus, PpVα peptide analogs induced (1) blockade of calcium channels in a time- and concentration-dependent manner, which was reversible after washout; (2) a reversible block of the voltage-dependent Na^+^ currents; (3) no block of the voltage-dependent Kv channels and interruption of K^+^ currents; (4) a drastic alteration of intracellular calcium. These results suggest that the neurotoxic action evoked by the three PpVα peptide analogs may be associated with alteration of cellular excitability by blocking the ionic currents responsible for the neurotransmitter release and cellular excitability. Neurotoxic peptides that modulate ion channels are emerging as molecular probes and lead compounds, expanding the arsenal of substances for diagnosing and treating degenerative neurological disorders, particularly those in which ion channels and receptors play essential roles. Finally, the present electrophysiological data warrant ongoing research involving these designed peptides as sequence scaffolds to dissect ion channel functions. Moreover, analogs of PpVα peptides can help elucidate the molecular basis of channelopathies.

Although PpVα peptides block both Na^+^ and Ca^2+^ channels, this activity reflects a selective and mechanistically coherent action rather than nonspecific interactions. Electrophysiological and molecular modeling data show that they preferentially target Nav1.7 and Cav2.2 channels, with minimal effect on K^+^ currents. The resulting modulation of intracellular calcium and oxidative stress is a physiologically relevant consequence of this targeted activity. These dual effects underpin the observed neuroprotective and cytoprotective outcomes, supporting the therapeutic potential of PpVα peptides in neurological disorders.

A molecule with these characteristics has significant clinical potential in various medical areas due to its ability to modulate neuronal activity and protect cells from oxidative damage. Some possible clinical uses involve: (i) Treatment of neurological diseases as epilepsy by blocking calcium and sodium channels could reduce neuronal hyperexcitability, a key feature of epileptic seizures; it could protect neurons from oxidative damage associated with chronic inflammation and axonal degeneration in multiple sclerosis; on in neurodegenerative diseases such as Alzheimer’s or Parkinson’s, where oxidative stress and neuronal dysfunction play an important role. (ii) Neuroprotection in acute events, such as stroke, by blocking sodium and calcium channels that could limit the excitotoxic cascade caused by excessive stimulation of NMDA receptors, reducing neuronal damage; or in traumatic brain or spinal cord injuries, where its neuroprotective action could decrease the extent of secondary damage mediated by oxidative stress. (iii) Management of chronic pain, such as neuropathic pain, where sodium and calcium channels are key in pain transmission. Blocking these channels could reduce the hyperactivity of pain pathways, providing relief in conditions such as postherpetic neuralgia or diabetic neuropathy. (iv) Prevention of ischemic damage in the context of ischemia–reperfusion (such as in cardiac surgeries or transplantation), where protection against oxidative stress could preserve the viability of sensitive neurons and other tissues. (v) Psychiatric disorders, such as anxiety and bipolar disorder, where this molecule could have a similar profile to some drugs that block ionic channels, such as lamotrigine, which have proven effective in mood disorder management.

In summary, the block of calcium channels can reduce excessive calcium entry into the cell, lowering the activation of enzymes that cause cellular damage. The block of sodium channels can stabilize the neuronal membrane, reducing the abnormal propagation of electrical impulses. The antioxidant action of these drugs could prevent damage to lipids, proteins, and DNA caused by reactive oxygen species (ROS), which are crucial in inflammation and cell death. Thus, the combination of these properties makes these peptide molecules promising in areas of high medical need, particularly in diseases characterized by excitotoxicity, inflammation, and oxidative stress.

## 4. Materials and Methods

### 4.1. Peptides

The structural features of the PpVα peptide analogs used in this study for the electrophysiology and neuroprotective experiments are shown in Table 1. The peptides were synthesized by solid-phase chemistry using a custom peptide service (China Peptides, Shanghai, China). The single internal disulfide bond in the folded peptide analog was formed in diluted aqueous solution by air-oxidization at basic pH. Analytical reverse-phase high-performance liquid chromatography and mass spectrometry analysis confirmed the purity grade (>95%) of the peptides and the presence of a single peak (see the Supplementary Material). The lyophilized peptides and peptide stock solutions (1 mM, in deionized water) were stored at −20 °C until use.

### 4.2. Three-Dimensional Structure Modeling and Molecular Dynamics Simulation of Peptides

The three-dimensional (3D) structures of the PpVα peptide analogs—PPA (Ppa-aSVP), SSB (SSb-aSVP), and CHI (Chi-aSVP)—were constructed using the AlphaFold2 server [56]. After the initial modeling, the candidate peptides’ 3D structures were submitted to CHARMM-GUI for C-terminal amidation and disulfide bond formation. These modified structures were then subjected to molecular dynamics simulations using the GROMACS 2018.6 software suite. The simulation setup involved several preparatory steps in CHARMM-GUI, including the construction of a water box around the peptides using the CHARMM36m All-Atom Force Field [57,58]. The system was solvated using a TIP3P water model, followed by energy minimization over 5000 steps. Subsequent NVT equilibration was conducted at a stable temperature of 310 K. The simulation’s production phase spanned 150 ns, using a 2-femtosecond timestep under NPT conditions to maintain a constant temperature of 310 K. The stability and conformational changes in the peptides were monitored by calculating root-mean-square deviation (RMSD), with the results graphically represented in QtGrace (version 0.26). Visualization of the 3D structures was performed using Discovery Studio (version 21.1.0).

### 4.3. Molecular Dynamics Simulation of Peptide-Protein Complexes

The crystallographic structures of the Na^+^ channel receptor (PDB ID: 5EK0) and the Ca^2+^-channel receptor (PDB ID: 7VFU) were obtained from the Protein Data Bank. These structures were submitted to the ZDOCK server for molecular docking with the PpVα peptide analogs, and ZRANK was used to evaluate the docking complex scores [59]. The peptide-protein interactions were analyzed using the Protein-Ligand Interaction Profiler (PLIP, https://plip-tool.biotec.tu-dresden.de/plip-web/plip/index, accessed one 23 September 2025), and the binding poses were visualized using PyMOL (version 2.3.0). Given that the Na^+^ and Ca^2+^-channel receptors are transmembrane proteins and the lipid bilayer influences the function and structure of these proteins, the peptide-protein complexes were subjected to CHARMM-GUI for membrane construction. The molecular dynamics (MD) simulation production phase was also run for 150 ns using the CHARMM36m All-Atom Force Field with GROMACS 2018.6, employing a 2-femtosecond timestep. The root-mean-square deviation (RMSD), the radius of gyration (Rg), and the number of hydrogen bonds were determined to assess the stability and conformational properties of the complexes.

### 4.4. Isolation and Culture of Bovine Chromaffin Cells

The care and use of animals followed the guidelines of the National Council on Animal Care and the European Communities Council Directive (86/609/ECC), and were approved by the local Animal Care Committee of Universidad Autónoma de Madrid (ES280790000092).

Chromaffin cells, like sympathetic neurons, develop from the neural crest. They are excitable cells with neuron-like electrical properties [37,60] with the capacity to synthesize, store, and release adrenaline and noradrenaline (for review, see [38]). They are among the most popular and widely used cellular models for investigating the molecular mechanisms underlying cellular excitability and neurotransmitter release [49,50,61].

In line with bioethical animal welfare practices and European regulations (EC-Nº 1099/2009), Spanish legislation requires a procedure that minimizes the animal’s pain and suffering until its death. The adrenal glands were from a local slaughterhouse under the supervision of the local veterinary service. For the stunning and slaughter of the animal, a pneumatic gun actuated by a captive bullet cartridge is used. The end of the barrel is attached to the animal’s skull, and it is fired. Bleeding by cutting the skin with a knife begins immediately after stunning. Bovine chromaffin cells were isolated by digestion of the adrenal medulla with collagenase. Two adrenal glands, which were grouped before plating, were used for each primary culture. Briefly, cells were suspended in Dulbecco’s modified Eagle’s medium (DMEM) and supplemented with 5% fetal bovine serum, 50 IU/mL penicillin, and 50 μg/mL streptomycin. Proliferation inhibitors (10 μM cytosine arabinoside, 10 μM fluorodeoxyuridine, and 10 μM leucine methyl ester) were added to the medium to prevent excessive fibroblast growth. Cells were plated on 1 cm-diameter glass coverslips for low-density patch-clamp studies (5 × 10^4^ cells per coverslip). Cells were plated at a density of 2 × 10^5^ cells per well in 96-well plates for intracellular calcium measurements. Cultures were maintained in an incubator at 37 °C in a water-saturated environment with 5% CO_2_. Cells were used 1–4 days after plating.

### 4.5. Electrophysiological Recordings

Voltage-clamp recordings were obtained using the whole-cell patch-clamp technique. Recordings were made using patch pipettes of thin fire-polished borosilicate glass (Kimax 51, Witz Scientific, Holland, OH, USA) to obtain a final series resistance of 2−5 MΩ when filled with the standard intracellular solutions and mounted on the head stage of an EPC-10 patch-clamp amplifier (HEKA Electronic, Lambrecht/Pfalz, Germany), allowing cancellation of capacitive transients and compensation of series resistance. Data were acquired with a 5–10 kHz sampling frequency and filtered at 1–2 kHz. Recording traces with leak currents >100 pA or series resistance 20 MΩ were discarded. The P/4 protocol was used to discard linear leaks and capacitive components. Data acquisition and analysis were performed using PULSE 8.74 software v 1.3 (HEKA Electronic, Lambrecht/Pfalz, Germany).

Coverslips containing 5 × 10^4^ cells were placed on a chamber mounted on the stage of a Nikon Eclipse T2000 inverted microscope (Leiden, The Netherlands). During the seal formation with the patch pipette, the chamber was continuously perfused with a control Tyrode solution containing (in mM) 137 NaCl, 5 KCl, 1 MgCl_2_, 2 CaCl_2_, 10 HEPES/NaOH (pH 7.4). Once the patch membrane was perforated and the whole-cell configuration of the patch-clamp technique was established, the cell was locally, rapidly, and constantly superfused with an extracellular solution of similar composition to the chamber solution but containing nominally 0 mM Ca^2+^ (to measure I_Na_), 10 mM Ca^2+^ (to measure I_Ca_) and 2 mM Ca^2+^ (to measure I_K_) (see Results for specific experimental protocols). For I_Na_ and I_Ca_ recordings, cells were dialyzed with an intracellular solution containing (in mM) 10 NaCl, 100 CsCl, 14 EGTA, and 20 TEA.Cl, 5 Mg-ATP, 0.3 Na-GTP, 20 HEPES/CsOH (pH 7.3). Cells were internally dialyzed with the internal solution to register IK, and CsCl and TEA were replaced by KCl. Tetrodotoxin (TTX, 5 μM) was added to the external solution to measure I_Ca_, and 200 μM Cd^2+^ was used to measure I_K_.

External solutions were rapidly exchanged using electronically driven miniature solenoid valves (The Lee Company, Westbrook, CO, USA) coupled to a multi-barrel concentration-clamp device, with the standard outlet placed within 100 µm of the cell to be patched. The flow rate was 1 mL/min. All the experiments were performed at room temperature (22–24 °C). Only one chromaffin cell was used for a single experiment.

Data were acquired using the PULSE 8.74 software (HEKA Elektronik, Lambrecht, Germany), with a sampling frequency of 20 kHz. The leakage current and capacitive components were removed using a P4 protocol, and the series resistance was compensated to 80%. The data analysis was performed using Igor Pro (Wavemetrics, Lake Oswego, OR, USA), PULSE (HEKA Elektronik), and Origin 8.0 (Microcal).

### 4.6. Measurements of [Ca^2+^]_c_ with Fluo-4-AM

These experiments used the fluorescent probe Fluo-4-AM (Thermo Fisher Scientific) and a microplate reader Fluostar Optima (BMG Labtech, Offenburg, Germany). After removing the medium, cells were incubated with the Ca^2+^ fluorescent probe Fluo-4 (Gibco-Invitrogen) (solution containing (in mM): 5.9 KCl, 144 NaCl, 1.2 MgCl_2_, 11 glucose, 10 HEPES/NaOH (pH 7.4), in which 10 μM fluo-4-AM and 0.2% pluronic acid were included) for 45 min at 37 °C in the dark. After this incubation period, cells were washed twice in the dark with Krebs-HEPES buffer at room temperature. Fluorescence measurements were performed using an excitation wavelength of 488 nm and an emission wavelength of 522 nm. At the end of the experiment, cells were incubated with Triton X-100 (5%) for 10 min to determine the maximum fluorescence (Fmax) and with MnCl_2_ (2 M) for 10 min to measure the minimum fluorescence (Fmin). Changes in [Ca^2+^]_c_ were calculated as a percentage of the total fluorescence; Fx = (F_measured_ − F_basal_)/(F_max_ − F_min_) × 100. All experiments were performed at room temperature on cells cultured for 1 to 3 days.

### 4.7. Cell Viability Assay with Human Neuroblastoma (SH-SY5Y) Cells

Chromaffin cells are commonly used in studies of exocytosis and the release of neurotransmitter. In contrast, neuroblastoma lines tend to be more robust in culture and better withstand stressful experimental conditions, such as the use of toxic agents or antioxidants in cytoprotection experiments.

SH-SY5Y cells derived from human neuroblastoma were used as experimental models for neuroprotection experiments. Being a subline of SK-N-SH cells, this cell line exhibits activity of choline acetyltransferase, acetylcholinesterase, dopamine-β-hydroxylase, and tyrosine, in addition to noradrenaline release [62]. These cells, aliquoted and frozen in liquid nitrogen, were suspended in DMEM/F-12 medium supplemented with 10% SBF, 50 IU/mL penicillin, and 50 μg/mL streptomycin and cultured in cell culture bottles. Once its proliferation and confluence have been achieved (usually 24–48 h after culture, the cells express the most characteristic phenotype for the assay). The cells were stored for 48 h at 37 °C in a saturated-humidity atmosphere containing 5% CO_2_ before use.

For fluorescence assays, the cells were treated with 0.25% Trypsin-EDTA (1 mL) to detach them from the support, then seeded into 96-well black flat-bottom plates at a density of 4 × 10^4^ cells/well. The cells were preserved for 24 h at 37 °C in an atmosphere saturated with humidity and 5% CO_2_. 24 h after planting, when the cells were attached to the surface, the experiment began and lasted for 3 days. In each plate, the experiment for each peptide and pharmacological substance solution was conducted in triplicate. Briefly, 200 μL of waste culture medium was removed from each well, and 200 μL of the fresh-prepared serum-free culture medium was added. The cells were exposed only to the treatment or the positive control. Within 48 h of plating, the wells were refilled. The solutions in the even wells were replaced with fresh solutions, while those in the odd wells included 200 μL of oligomycin/rotenone (O/R) (cell death inducers). 72 h after planting, 200 μL of 10% resazurin (which allows detection of cell viability by converting a non-fluorescent stain (blue) to a fluorescent form (pink); the signal detected will be proportional to the number of living cells) was added to each well. After 5 h, cell viability was measured. Fluorescence intensity, and therefore cell viability, was measured using a multimode plate reader (FLUOstar).

### 4.8. Statistical Analysis

No blinding or sample size calculations were performed in the study. The sample size was estimated and verified to have sufficient power, with alpha and beta risks set at 0.05 and 0.2, respectively, in a two-sided test. Four subjects are necessary in the first group (control) and 4 in the second (experimental condition) to recognize a difference greater than or equal to 2 units as statistically significant. The typical standard deviation is assumed to be 1. It has been anticipated that the dropout rate will be 0%. Data were expressed as means ± SEM. of the number of experiments performed (n) from at least three cell cultures. Students’ *t*-tests or one-way ANOVAs followed by Dunnett’s multiple comparisons test were used to determine statistical significance between means. Statistical significance was established at *p*-values < 0.05 (*), < 0.01 (**), and < 0.001 (***). No data points were excluded, and no test for outliers was performed. All analyses were performed using GraphPad Prism 8.01.

A limitation of the present study is that the investigators were not blinded to the experimental conditions during data collection and analysis. This may introduce the potential for experimenter bias. Future studies will address this issue by implementing blind experimental designs to ensure greater objectivity and reproducibility.

All datasets were examined for consistency and data integrity prior to analysis. No extreme or inconsistent values were detected; therefore, no outlier removal procedure was applied.

### 4.9. Chemicals

Collagenase type I was obtained from Roche laboratory (Madrid, Spain), while Dulbecco’s Modified Eagle Medium (DMEM), fraction V fetal bovine albumin, and penicillin-streptomycin were from Gibco (Thermo Fisher Scientific, Madrid, Spain). Fluo-4-AM was from Molecular Probes (Thermo Fisher Scientific, Madrid, Spain), and the rest of the chemical reagents and solutions were from Sigma, Merck, and Panreac Chemical (Madrid, Spain).

## 5. Conclusions

The conclusion of this study can be emphatically summarized as highlighted points, as follows:

- PpVα peptide analogs induced a partially reversible block of calcium channels in a time- and concentration-dependent manner.
- PpVα peptide analogs caused a reversible block of the voltage-dependent Na^+^ currents at the micromolar range.
- PpVα peptide analogs did not block the voltage-dependent K^+^ currents.
- PpVα peptide analogs produced a drastic alteration of intracellular calcium.

## Figures and Tables

**Table 1:** Sequences and structural characteristics of PpVα peptide and analogs in this study.

Peptide	Primary Sequence and Predicted Model	Obs.	Theoretical MW ^a^	Experimental MW ^b,^*
PpVα (PPA)	KYWILNVPASVCDEYCWSQMLLYKKS-NH_2_ 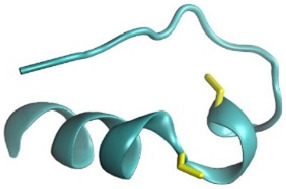	Linear, helical 26-residue peptide	3167.73	3167.77
Folded (SSB)	KYWILNVPASVC_12_DEYC_16_WSQMLLYKKS-NH_2_ 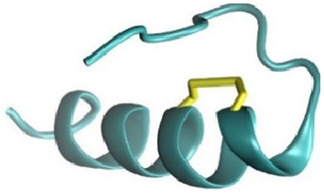	Internal C12-C16, S-S bond	3165.71	3165.77
Chimeric (CHI)	GELIKMKYWILNVPASVCDEYCWSQMLLYKKS-NH_2_ 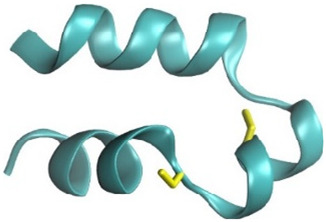	Fused sequence; additional six N-terminal residues	3837.56	3839.64

Notes: ^a^ The theoretical molecular weight (MW) was calculated using PepCalc (https://pepcalc.com/, (accessed on 23 September 2025); ^b^ The experimental MW was determined from the MS spectra of purified peptides. The predicted models were generated with the PEP-FOLD server (https://bioserv.rpbs.univ-paris-diderot.fr/services/PEP-FOLD/, (accessed on 23 September 2025). Carboxyl-terminus (C-terminal) α-helices are visible in the linear PpVα (PPA) and in the folded (disulfide-bonded) SSB peptide with the unstructured N-terminal stretches. The chimeric PpVα peptide contains an extra fused α-helix at the N-terminal six-residue stretch with a similar homologous Protopalythoa variabilis peptide sequence. Cysteine residues and disulfide bonds are in yellow. PPA = Protopalythoa V-shape alpha helical peptide. SSB = single disulfide bound, Protopalythoa V-shape alpha helical peptide. CHI = chimeric Protopalythoa V-shape alpha helical peptide. * MS spectra are available in the Supplementary Material.

**Table 2:** Neuroprotective effect of PpVα peptide analogs in reversing the neurotoxicity caused by veratridine in neuroblastoma cells. The data represent the mean values.

Peptide	Cell Viability (%)					
	Control	VTD	TTX	1 μM	3 μM	10 μM
	100	49.21	85.41			
PPA				69.29	80.45	81.88
SSB				85.32	93.23	99.40
CHI				78.38	81.25	74.03

Notes: VTD, veratridine; TTX, tetrodotoxin; PPA, *Protopalythoa variabilis* V-shape helical peptide (PpVα); SSB, Single disulfide bond, folded PpVα; CHI, chimeric PpVα.

**Table 3:** Neuroprotection of neuroblastoma (SH-SY5Y) cells against oligomycin A and rotenone (O/R)-induced mitochondrial stress. The data represent the mean values.

Peptide			Cell Viability (%)			
	Control	O/R	MLT	1 μM	3 μM	10 μM
	100	39	82			
PPA				77	71	60
SSB				75	68	65
CHI				71	73	67

**Notes:** O/R, oligomycin and rotetone; MLT, melittin; PPA, *Protopalythoa variabilis* V-shape helical peptide (PpVα) SSB, Single disulfide bond, folded PpVα; CHI, chimeric PpVα.

## Data Availability

The original contributions presented in this study are included in the article/supplementary material. Further inquiries can be directed to the corresponding authors.

## Supplementary Materials

The following supporting information can be downloaded at: https://www.mdpi.com/article/10.3390/ph18111701/s1.
