# Supplementary material for: Inhibitory Activity of Calcium and Sodium Ion Channels of Neurotoxic Protoplaythoa variabilis V-Shape Helical Peptide Analogs and Their Neuroprotective Effect In Vitro
Source: Pharmaceuticals (Basel). 2025 Nov 10;18(11):1701. doi: 10.3390/ph18111701 (PMC12655438; doi:10.3390/ph18111701)
Supplement: Supplementary file 1 [file pharmaceuticals-18-01701-s001.zip › pharmaceuticals-3920039-supplementary.pdf]

Peptide Name: PpVα (PPA)

Sequence: KYWILNVPASVCDEYCWSQMLLYKKS-NH2

Sequence (Three Letters Code): Lys-Tyr-Trp-Ile-Leu-Asn-Val-Pro-Ala-Ser-Val-Cys-Asp-Glu-Tyr-Cys-Trp-Ser-Gln-Met-Leu-Leu-Tyr-Lys-Lys-Ser-NH2

Molecular Weight: 3167.77

HPLC Analysis: Peptide Purity:97.08%

(See attached RP-HPLC chromatogram)

MS Analysis: ESI_MS

(See attached MS spectrum)

Solubility: 1mg/ml in 30%ACN/70%H_2O

Appearance: lyophilized powder

Counter Ion: Trifluoroacetate

Remarks:

HPLC Analysis Report

Measurement:	Peak Area	Run Time:	16min
Calculation Type:	Percent	Wavelength:	220nm
Flow Rate:	1ml/min	Inj.Vol:	10uL
Buffer A:	0.1% TFA in water	Buffer B:	0.1%TFA in Acetonitrile
Column:	Kromasil 100-5C18,4.6mmX250mm,5 micron	Column Temp:	35℃
Gradient(linear):	35%-75% buffer B in 16min		

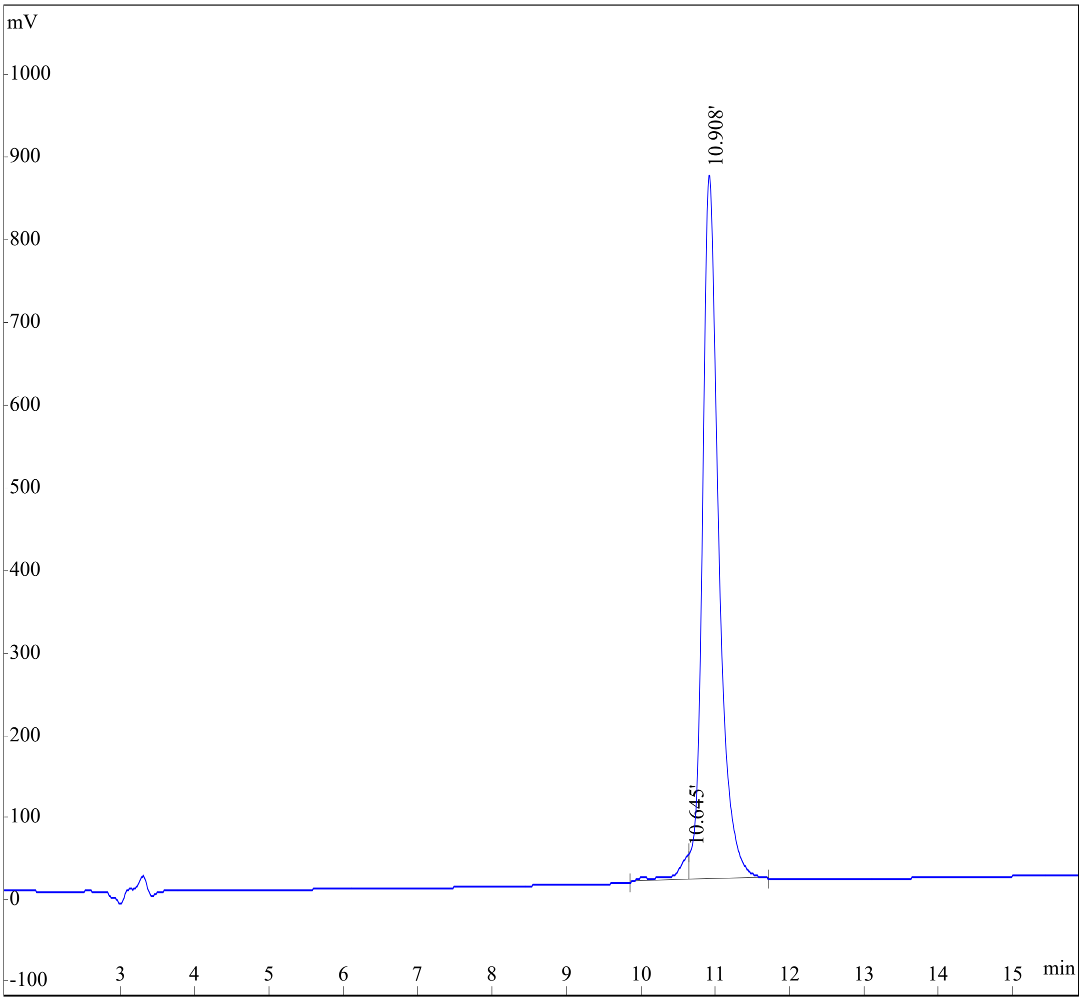

Rank	Time	Name	Conc.	Area
1	10.645		2.925	391045
2	10.908		97.08	12977678
Total			100	13368723

MS Analysis Report

Expected MS: 3167.77

Ion Source: ESI

NEB:10.00 CUR:12.00

Flow Rate : 0.2ml/min

Mass Spectrometer: API 150EX

B.conc: 75%ACN/24.5%H_2O/0.5%Ac

IS:+4500 TEM:0.00

Run Time: 1min

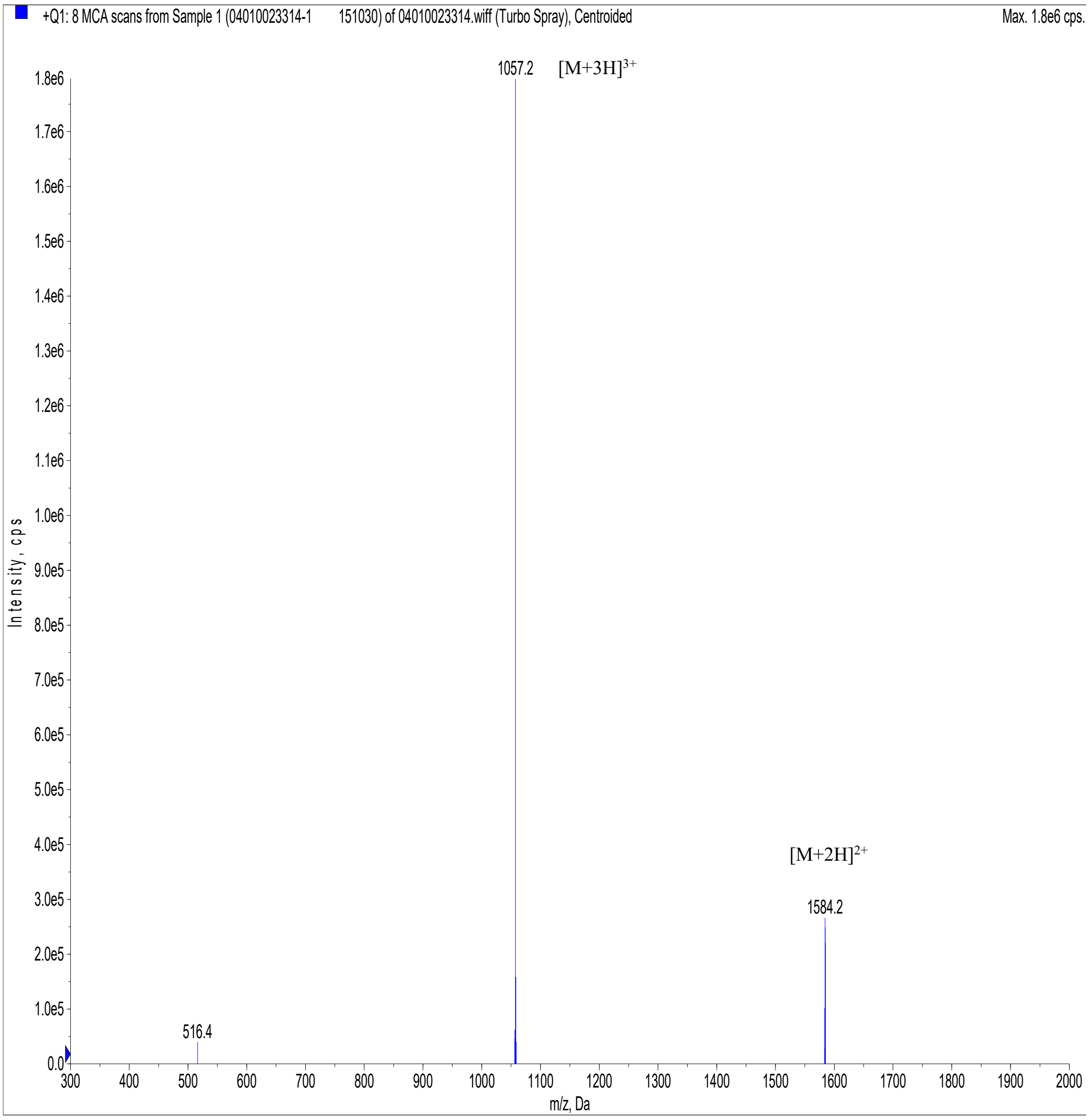

Product Name: Folded PpVα (SSB)

Sequence: KYWILNVPASVCDEYCWSQMLLYKKS-NH2
(Disulfide bond C12-C16)

Sequence (Three Letters Code): Lys-Tyr-Trp-Ile-Leu-Asn-Val-Pro-Ala-Ser-Val-Cys-Asp-Glu-Tyr-Cys-Trp-Ser-Gln-Met-Leu-Leu-Tyr-Lys-Lys-Ser-NH2 (Disulfide bond C12-C16)

Molecular Weight: 3165.77

HPLC Analysis: Peptide Purity:95.94%

(See attached RP-HPLC chromatogram)

MS Analysis: ESI_MS

(See attached MS spectrum)

Solubility: 1mg/ml in 30%ACN/70%H_2O

Appearance: lyophilized powder

Counter Ion: Trifluoroacetate

HPLC Analysis Report

Measurement:	Peak Area	Run Time:	17min
Calculation Type:	Percent	Wavelength :	220nm
Flow Rate :	1ml/min	Inj.Vol:	10uL
Buffer A :	0.1% TFA in water	Buffer B:	0.1%TFA in Acetonitrile
Column:	Kromasil 100-5C18,4.6mmX250mm,5 micron	Column Temp:	35℃
Gradient(linear):	20%-62% buffer B in 17min		

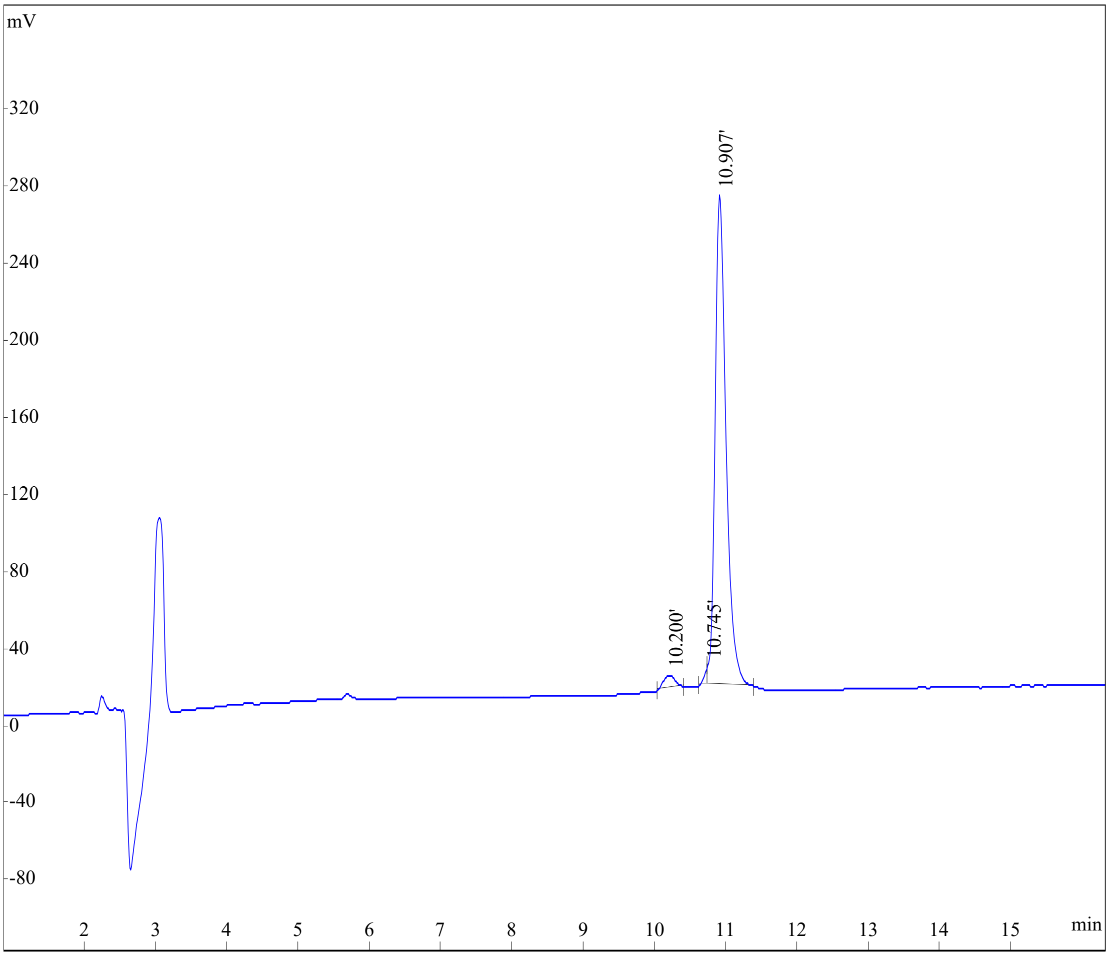

Rank	Time	Name	Conc.	Area
1	10.200		2.876	81498
2	10.745		1.182	33487
3	10.907		95.94	2718649
Total			100	2833634

MS Analysis Report

Expected MS: 3165.77

Ion Source: ESI

NEB:10.00 CUR:12.00

Flow Rate : 0.2ml/min

Mass Spectrometer: API 150EX

B.conc: 75%ACN/24.5%H_2O/0.5%Ac

IS:+4500 TEM:0.00

Run Time: 1min

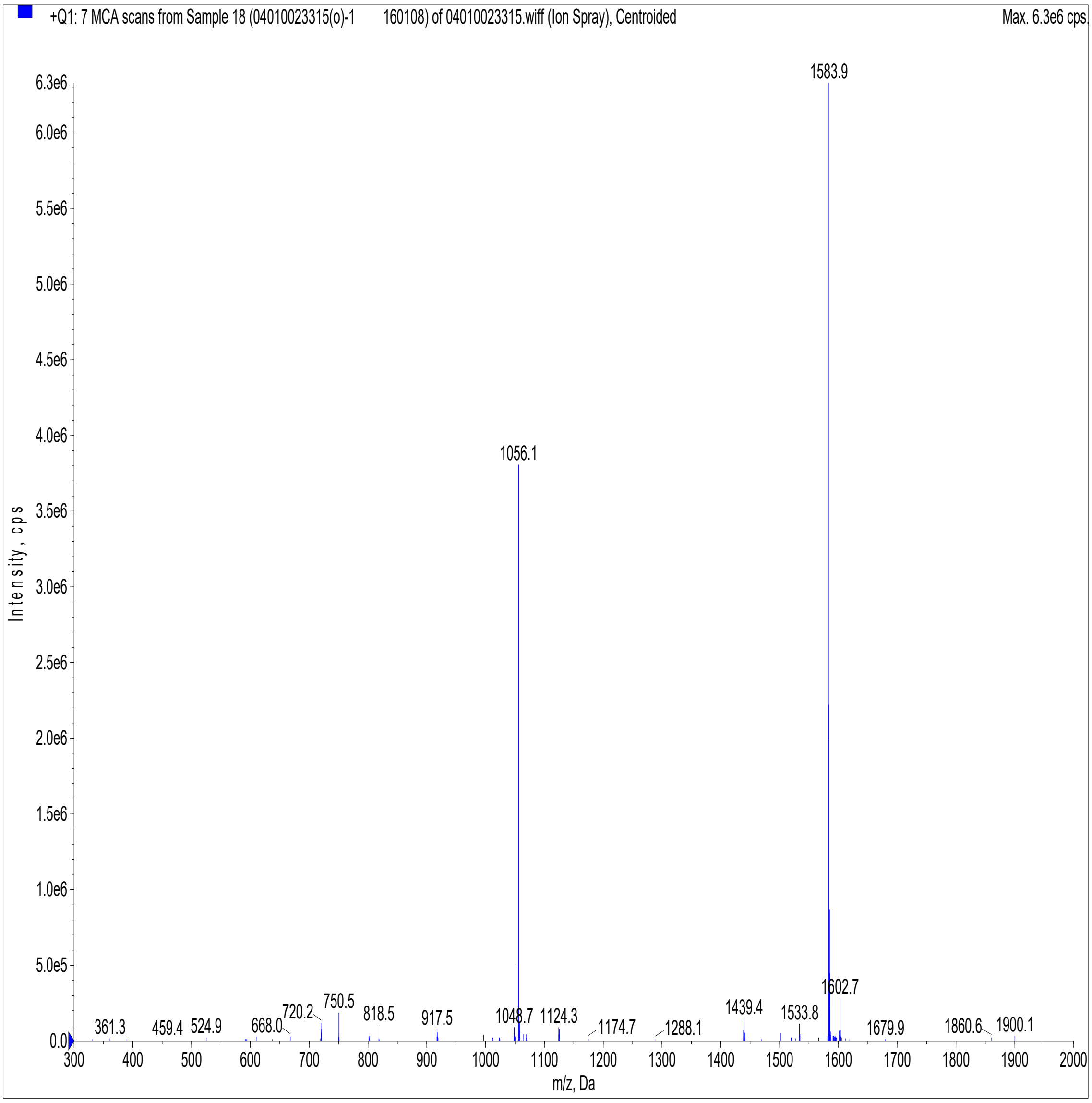

**Product Name:**	Chimeric PpVα (CHI)
**Sequence:**	GELIKMKYWILNVPASVCDEYCWSQMLLYKKS-NH2
**Sequence (Three Letters Code):**	Gly-Glu-Leu-Ile-Lys-Met-Lys-Tyr-Trp-Ile-Leu-Asn-Val-Pro-Ala-Ser-Val-Cys-Asp-Glu-Tyr-Cys-Trp-Ser-Gln-Met-Leu-Leu-Tyr-Lys-Lys-Ser-NH2
**Molecular Weight:**	3839.64
**HPLC Analysis:**	Peptide Purity:96.06%
	(See attached RP-HPLC chromatogram)
**MS Analysis:**	ESI_MS
	(See attached MS spectrum)
**Solubility:**	1mg/ml in 20%ACN/80%H_2O
**Appearance:**	lyophilized powder
**Counter Ion:**	Trifluoroacetate

HPLC Analysis Report

Measurement:	Peak Area	Run Time:	14min
Calculation Type:	Percent	Wavelength :	220nm
Flow Rate :	1ml/min	Inj.Vol:	10uL
Buffer A :	0.1% TFA in water	Buffer B:	0.1%TFA in Acetonitrile
Column:	Kromasil 100-5C18,4.6mmX250mm,5 micron	Column Temp:	35℃
Gradient(linear):	20%-55% buffer B in 14min		

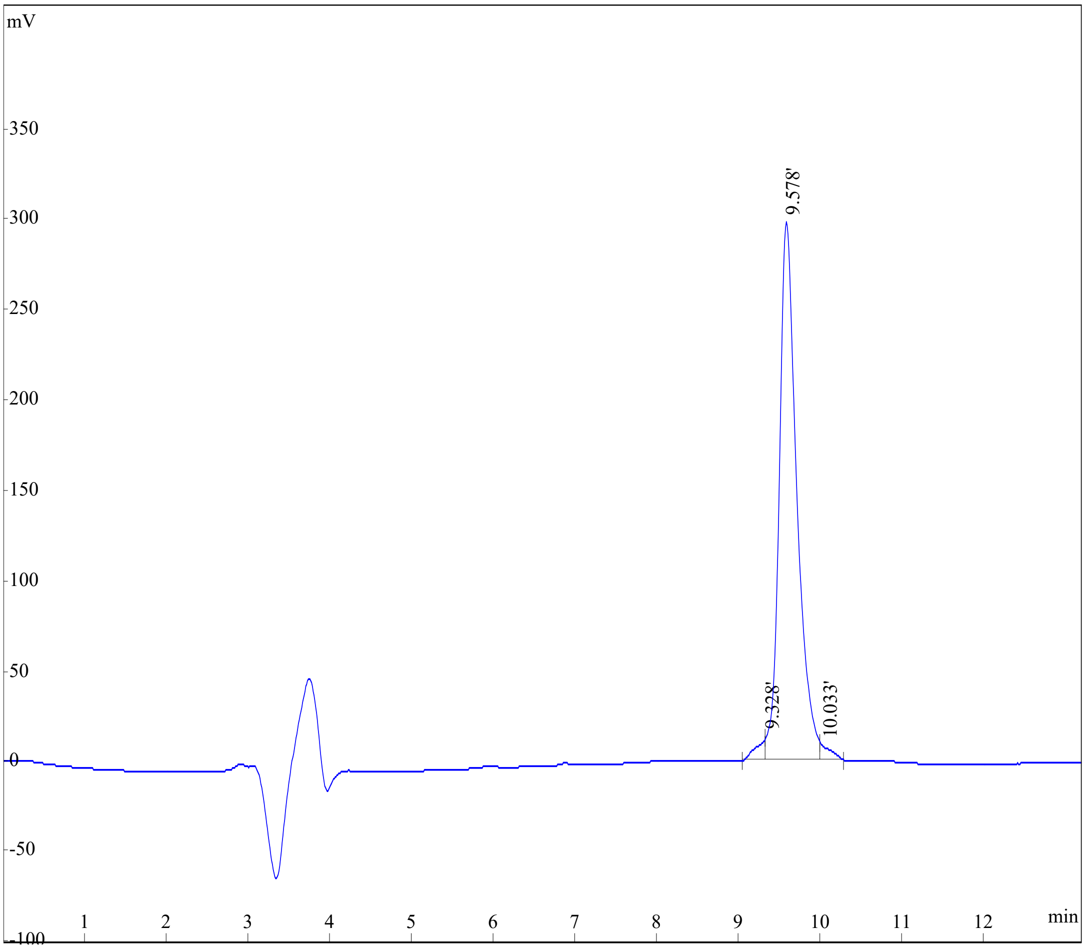

Rank	Time	Name	Conc.	Area
1	9.328		2.237	104240
2	9.578		96.06	4476560
3	10.033		1.709	79644
Total			100	4660444

MS Analysis Report

Expected MS: 3839.64	Mass Spectrometer: API 150EX
Ion Source: ESI	B.conc: 75%ACN/24.5%H_2O/0.5%Ac
NEB:10.00 CUR:12.00	IS:+4500 TEM:0.00
Flow Rate : 0.2ml/min	Run Time: 1min
